# Hepatitis B Virus Neutralization with DNA Origami Nanoshells

**DOI:** 10.1021/acsami.4c03700

**Published:** 2024-05-10

**Authors:** Elena M. Willner, Fenna Kolbe, Frank Momburg, Ulrike Protzer, Hendrik Dietz

**Affiliations:** ‡ Department of Biosciences, School of Natural Sciences and Munich Institute of Biomedical Engineering, Technical University of Munich, Boltzmannstraße 11, 85748 Garching, Germany; § Institute of Virology, School of Medicine & Health, Technical University of Munich and Helmholtz Munich, Trogerstraße 30, 81675 Munich, Germany; ∥ Translational Immunity Unit, German Cancer Research Center (DKFZ), Im Neuenheimer Feld, 69120 Heidelberg, Germany; ⊥ German Center for Infection Research (DZIF), Munich Partner Site, 81675 Munich, Germany

**Keywords:** DNA origami, hepatitis B virus, viral blocking, antivirals, *in vitro* neutralization

## Abstract

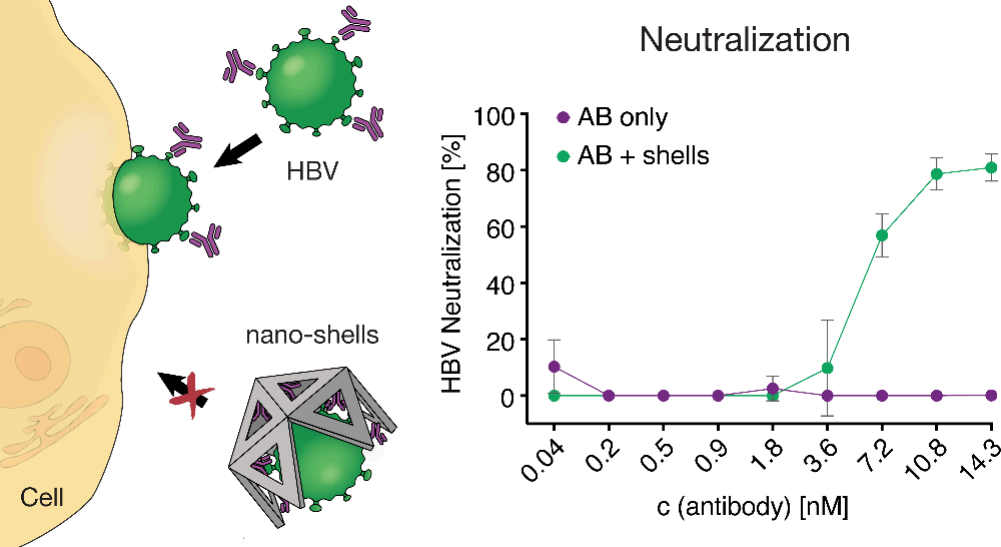

We demonstrate the use of DNA origami to create virus-trapping nanoshells that efficiently neutralize hepatitis B virus (HBV) in cell culture. By modification of the shells with a synthetic monoclonal antibody that binds to the HBV envelope, the effective neutralization potency per antibody is increased by approximately 100 times compared to using free antibodies. The improvements in neutralizing the virus are attributed to two factors: first, the shells act as a physical barrier that blocks the virus from interacting with host cells; second, the multivalent binding of the antibodies inside the shells lead to stronger attachment to the trapped virus, a phenomenon known as avidity. Pre-incubation of shells with HBV and simultaneous addition of both components separately to cells lead to comparable levels of neutralization, indicating rapid trapping of the virions by the shells. Our study highlights the potential of the DNA shell system to rationally create antivirals using components that, when used individually, show little to no antiviral effectiveness.

## Introduction

Programmable self-assembly with DNA origami allows for the creation of user-defined three-dimensional (3D) nanoscale structures. These structures can be used for a wide range of applications, including drug delivery, biosensing, and molecular motors, and as templates for synthesizing inorganic materials.^1−6^ The programmability of DNA origami enables precise control over the size, shape, and composition, making it a versatile platform for constructing nanoscale structures with tailored functionalities. In DNA origami, sets of DNA single strands (“staples”) are designed to base pair with a long single-stranded DNA molecule (“scaffold”) to fold it into a predefined shape.^7−9^ Multiple discrete DNA origami building blocks, in turn, can then oligomerize into well-defined higher order 3D objects^10^ with dimensions that can exceed those of viruses,^11^ and it has been explored using such shells and other DNA nanoarchitectures to inhibit the entry of viruses to cells.^12−16^ In principle, any moiety that binds viruses could be used to coat the inside of DNA origami nanoshells, regardless of whether it has inherent neutralizing properties. One distinctive aspect of our shells is their ability to remain effective even if the binders themselves are not neutralizing. The binders are used to capture the virus inside the nanoshells, which subsequently function as an entry inhibitor. In addition, the efficacy of virus binders with neutralization capacities is expected to be enhanced when used within the shells, because the shell material can contribute to blocking viruses from interactions with cells by presenting a physical barrier to infection. Furthermore, the density of the virus binders inside the DNA shells may be controlled by the user, so that avidity effects stemming from the simultaneous binding of multiple virus binders to the same virus can be elicited, which is also expected to enhance neutralization potency. In the present work, we test the neutralization capacity of DNA origami nanoshells, exemplarily in cell cultures using infectious and replicative hepatitis B viruses as a model system.

**Figure 1:**
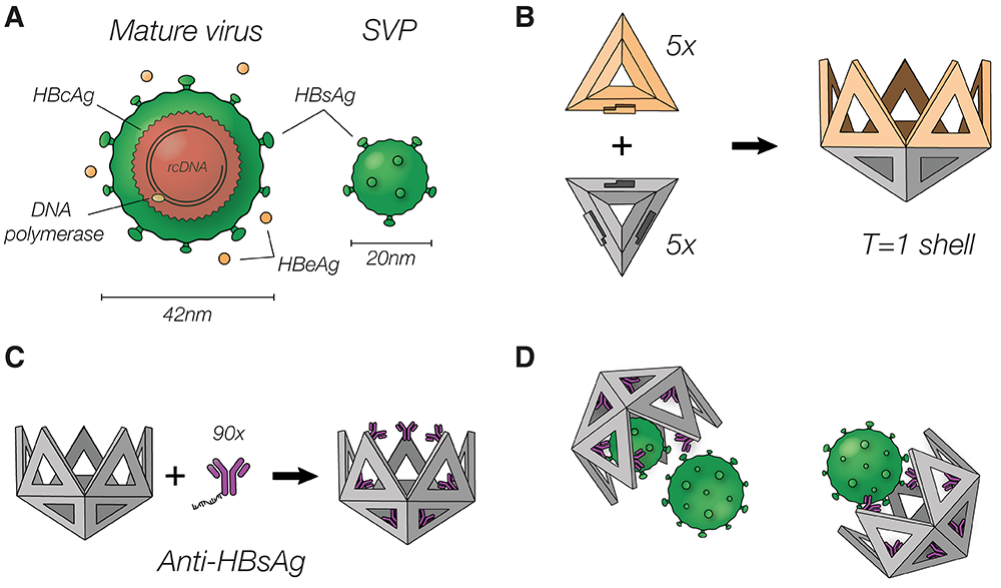
Hepatitis B virus overview and capture using DNA nanoshells. (A) Illustration of the hepatitis B virus. Left, the mature virus; right, the subviral particle (SVP) consisting of solely HBsAg. (B) Visualization of the *T* = 1 nanoshell (right) and its two different triangular subunits (left). Five gray triangles build the pentameric base, and five yellow triangles bind to this base to create a deeper cavity. (C) Functionalization of the *T* = 1 nanoshell: HBsAg antibodies are functionalized with a ssDNA strand and mounted to the 90 binding sites inside the shell. (D) Functionalized nanoshells can specifically recognize and bind HBV and its subviral particles.

The hepatitis B virus (HBV) is an enveloped virus of the *Hepadnaviridae* family. HBV predominantly infects hepatocytes and can cause significant liver damage, resulting in liver cirrhosis. It is the single most frequent cause of liver cancer, known as hepatocellular carcinoma, which is difficult to treat and one of the most deadly cancers.^17,18^ HBV is a spherical enveloped virus with an approximately 42 nm diameter (Figure 1A) containing an icosahedral capsid that encapsulates the viral DNA genome, which occurs in a relaxed circular form (rcDNA) and has a length of about 3200 bp.^19^ The capsid is a homopolymer built from 180 or 240 subunits of the HBV core protein (HBcAg; Figure 1A, red). The HBV rcDNA genome is formed in the viral capsid by a DNA polymerase with a reverse transcriptase activity using a 3.5 kb pregenomic RNA as a template. The envelope consists of a lipid bilayer membrane densely packed with the virus surface antigens (HBsAg), known as the large (L), medium (M), and small (S) surface proteins, with the latter being produced in excess.

After infection of a hepatocyte, rcDNA is transported to the nucleus and converted to the covalently closed circular DNA (cccDNA), which is the nuclear persistence form of HBV. HBV replication in the cell produces an additional antigen known as the hepatitis B e antigen (HBeAg; Figure 1A, yellow). HBeAg is secreted by the host cell and can be used as a marker of active viral replication.^20^ In addition to mature HBV virions, HBV-positive cells typically also secrete non-infectious spherical and tubular subviral particles mainly consisting of the outer envelope proteins S. These subviral particles are smaller than the actual HBV virions, with diameters of approximately 20–22 nm.^21^ The fully assembled hepatitis B viruses fit into a previously described DNA nanoshell prototype^12^ (Figure 1B). Na^+^–taurocholate co-transporting polypeptide (NTCP) expressing HepG2 cells present a reliable *in vitro* system to quantify HBV infection^22,23^ and infection neutralization.^24^ Markers, such as HBeAg and cccDNA, can be used to analyze the level of HBV infection. We used a synthetic antibody (MoMAb) that consists of two copies of a single-chain antibody fragment fused to a mutated fragment crystallizable (Fc) domain of immunoglobulin G1 (IgG1) with reduced Fc-receptor binding affinity^25^ to coat DNA nanoshells and test their neutralization capacity relative to that of the free antibodies.

## Results and Discussion

To trap hepatitis B virus particles, we created icosahedral DNA origami nanoshells with an 80 nm wide cavity (Figure 1B), using two distinct triangular building blocks as previously described.^12^ To stabilize the shells for cell culture, we covalently cross-linked the constituent triangular subunits using ultraviolet (UV) point welding.^26^ We included nine single-stranded DNA (ssDNA) handles that protrude from each of the triangular subunits, creating 90 attachment sites in the internal cavity of the shell. We selected a synthetic immunoglobulin G (IgG) antibody against HBsAg, referred to as MoMAb, as a binder^25^ and labeled it with thiolated DNA single strands that were complementary to the handles displayed on the interior surface of the shells. To tag the antibodies with DNA, we used a sulfosuccinimidyl 4-(*N*-maleimidomethyl)cyclohexane-1-carboxylate (sulfo-SMCC) cross-linker to target surface-exposed lysine residues on the IgG (Figures S2 and S3 of the Supporting Information). We then added the DNA-tagged MoMAb in 90:1 excess to a solution containing the DNA shells to populate all binding sites inside the shell cavity (Figure 1C). With this coating, we successfully trap HBV in DNA nanoshells (Figure 1D).

**Figure 2:**
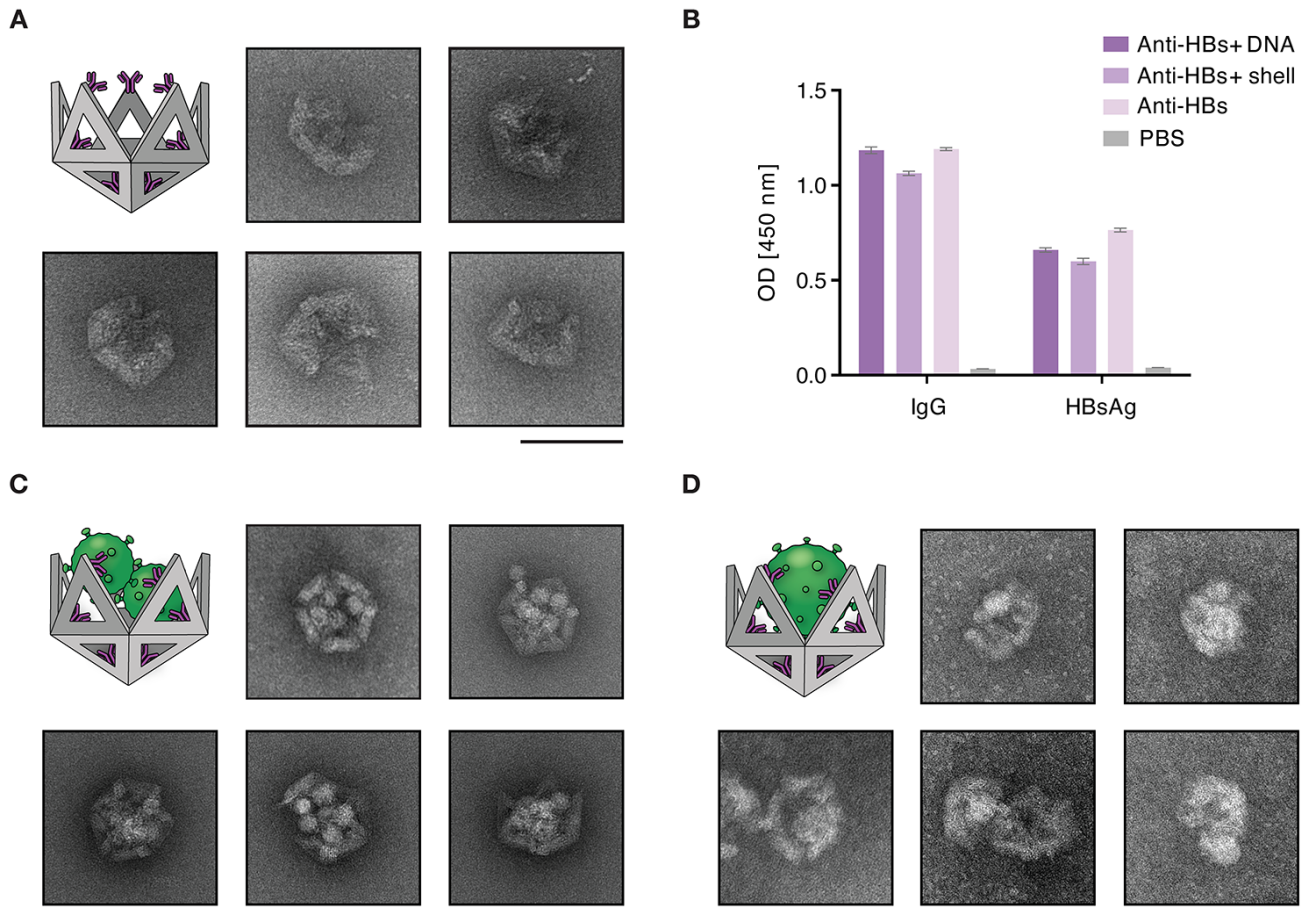
Shell functionalization and resulting HBV subviral particle and virion capture. (A) Negative stain TEM images of the functionalized DNA shell using the anti-HBs antibody MoMAb. (B) Binding control of the functionalized anti-HBs antibody. Nanoshells conjugated with MoMAb anti-HBs antibodies (medium violet) and with anti-HBs conjugated to the DNA handle without formation of nanoshells (dark violet) were analyzed by ELISA with either anti-IgG or HBsAg immobilized to the plates. MoMAb alone (light violet) served as a positive control, and PBS (gray) served as a negative control. (C and D) TEM images of the functionalized DNA shells capturing multiple (C) subviral particles and (D) HBV virions. HBV particles were fixed with formaldehyde prior to incubation with DNA nanoshells. The scale bar indicates 100 nm.

To ensure stability under cell culture conditions, we coated the antibody-functionalized nanoshells with a mixture of polylysine and polyethylene glycol–polylysine (PEG–polylysine),^27,28^ a stabilization strategy that has previously been shown to stabilize similar DNA nanoshells for at least 24 h.^12^ We confirmed the structural integrity using negative-staining transmission electron microscopy (TEM) (Figure 2A and Figure S4A of the Supporting Information). To test the ability of the functionalized nanoshells to trap HBV, we added non-infectious subviral particles to the shells. We imaged them using TEM, which revealed dense packaging of the subviral particles inside the shell cavity (Figure 2C and Figure S4B of the Supporting Information). We also attempted to trap purified infectious HBV virions using the shells. To this end, the HBV particles were fixed with paraformaldehyde and administered to the functionalized nanoshells. As a result of the low concentration of the purified, enveloped HBV virions that we obtained in comparison to subviral particles or non-enveloped viruses, TEM imaging proved challenging (Figure 2D and Figure S4C of the Supporting Information).

**Figure 3:**
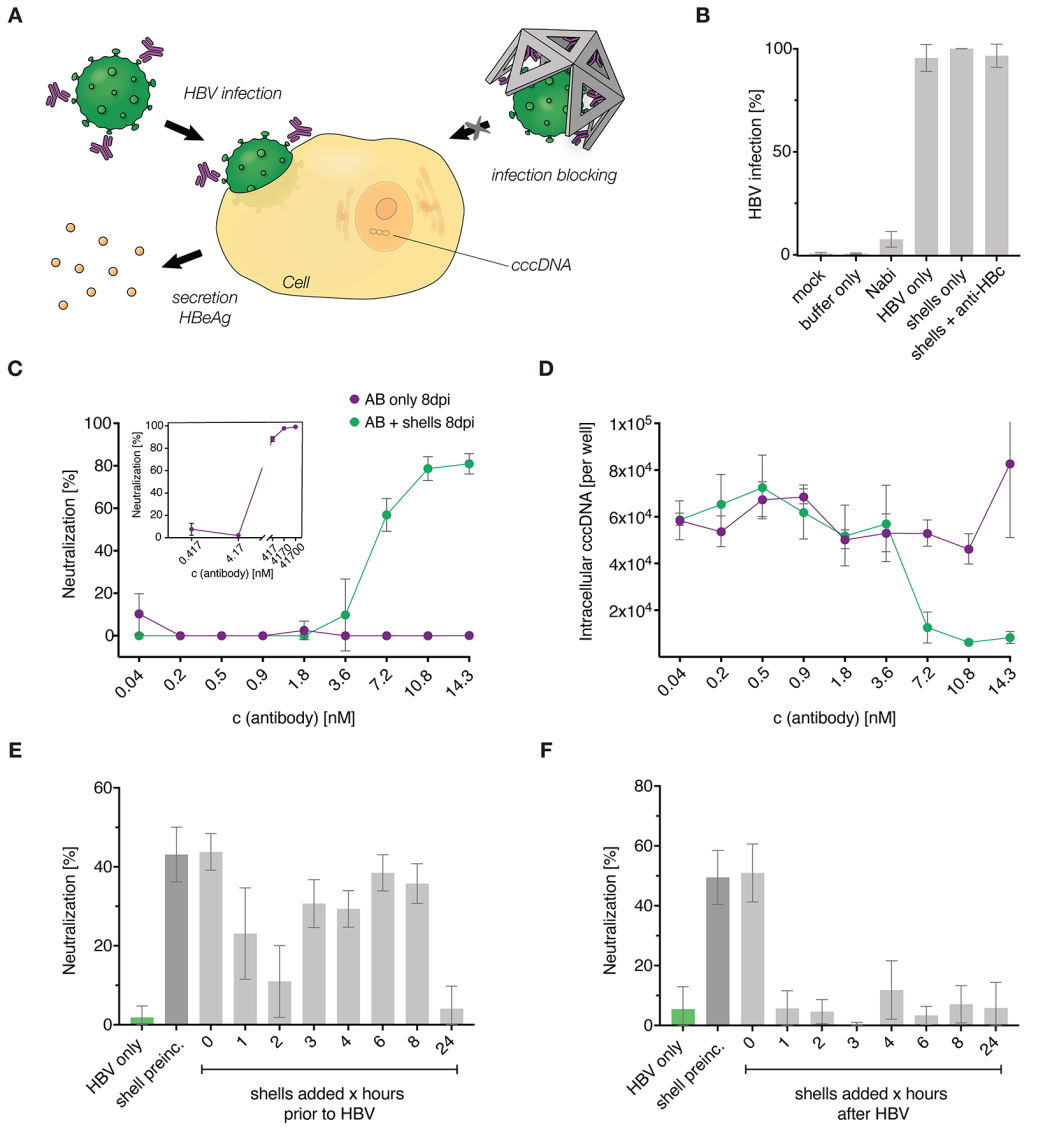
Hepatitis B virus neutralization. (A) Illustration of the neutralization assay comparing the neutralization capabilities of antibodies only versus DNA nanoshells functionalized with antibodies using wild-type HBV. If the host cell becomes infected, it starts secreting HBeAg (shown in yellow) and viral cccDNA can be found in the nucleus (indicated in orange). (B–D) NTCP expressing HepG2 cells were infected with wild-type HBV, and infection efficacy was analyzed 8 days after infection. (B) Infection efficacy determined by the relative HBeAg secretion level quantified by ELISA. The background was determined using the cell culture medium or the buffer used for nanoshell production. Infection neutralization by the therapeutically used human immune globulin Nabi-HB is shown in comparison to the positive control of adding HBV only, HBV with unfunctionalized nanoshells, or nanoshells functionalized with polyclonal anti-HBc antibody, which should not bind HBsAg. Neutralization capacity is given as a percentage based on HBeAg detection. (C) Neutralization capacity of nanoshells functionalized with MoMAb anti-HBs antibody (purple) compared to the free antibody (green). The inset shows the neutralization capacity of free antibody for a larger concentration range. Neutralization capacity is given as a percentage based on HBeAg detection via ELISA. (D) Intracellular HBV cccDNA quantified by PCR. (B–D) Data are represented as the mean ± standard deviation (sd) of *n* = 3 independent experiments. (E and F) HepG2-NTCP cells were infected with rHBV expressing Nanoluc luciferase. (E) Neutralization capacity of nanoshells administered to HepG2-NTCP cells at different time points before HBV infection. (F) Neutralization capacity of nanoshells administered to HepG2-NTCP cells at different time points after HBV infection. Shell preinc. indicates functionalized shells that were pre-incubated with HBV for 3 h before administration to the cells. Infection efficacy and levels of neutralization were determined by luciferase activity in cell culture medium. Respective values determined in mock-treated cells served to define 0% neutralization. The data are represented as the mean ± sd and are composed of *n* = 6 independent experiments.

To determine the ability of the DNA nanoshells to neutralize infectious HBV in cell culture, we carried out experiments schematically depicted in Figure 3A. We quantified the degree of cellular infection by measuring secreted HBeAg levels and intracellular cccDNA using enzyme-coupled immunosorbent assays (ELISAs) and quantitative polymerase chain reaction (q-PCR), respectively.

To normalize the data, we used the levels of these markers in control HBV infection samples, where HBV was allowed to infect and proliferate freely. The term “neutralization” denotes the fractional suppression of infection relative to those controls. We seeded NTCP-expressing HepG2 cells in a differentiation medium 3 days before infecting them with HBV at a multiplicity of infection (MOI) of 100 enveloped, DNA-containing virus particles per cell. The infectious HBV particles were pre-incubated with either DNA nanoshells or free antibodies for 3 h at 37 °C and then administered to the cells. After 18 h, we removed the inoculum, washed the cells, and added fresh media. Cell culture supernatants were collected 4 and 8 days post-infection, respectively, to determine HBeAg and cccDNA levels.

To establish the robustness of the assay and assess the specificity of the functionalized nanoshells, we performed several control experiments. Mock and buffer samples not containing any HBV showed a 0% infection rate and were used to define a hypothetical 100% infection neutralization rate. Multiple biological replicates of HBV-only controls were used to set a 100% infection rate within the experimental variation. Non-functionalized nanoshells were incubated at equivalent stoichiometric ratios to HBV and did not interfere with infection, which was anticipated because non-functionalized shell variants should not interact with HBV. Finally, we prepared a functionalized version of the nanoshells coated with an IgG antibody recognizing HBcAg; i.e., the capsid inside the virus particles that should not be accessible in the infectious and enveloped variant of HBV. Therefore, anti-HBc IgG functionalized nanoshells were expected to neither interact with the enveloped HBV nor suppress infection. As expected, we observed negligible infection suppression using this control (Figure 3B).

Next, we collected dose–response data for neutralization using both anti-HBs MoMAb alone and the nanoshells functionalized with this antibody (panels C and D of Figure 3). The data are presented as a function of the effective MoMAb IgG concentration to ensure a fair comparison. Because 90 IgG molecules were coated per shell, the actual shell concentration is 90 times lower than indicated on the graphs. Our results show that the MoMAb-functionalized nanoshells potently neutralized the viruses with an estimated half maximal inhibitory concentration (IC_50_) of ∼5 nmol/L in terms of effective IgG antibody concentration, equivalent to an IC_50_ of ∼55 pmol/L in terms of the actual DNA origami nanoshell concentration. When using free MoMAb IgG at 5 nmol/L instead of mounting them in groups of 90 on nanoshells, we observed negligible neutralization effects, with neutralization occurring only at ∼100-fold higher concentrations of around 0.5 μmol/L free IgG (inset in Figure 3C). We also quantified the amount of intracellular cccDNA in the cells indicating the number of infected cells (Figure 3D). The amount of intracellular cccDNA dropped sharply in the dose–response curves obtained for the DNA nanoshells at an effective IgG concentration of ∼5 nM, which is consistent with our findings with the HBeAg assay (Figure 3C).

To investigate how a temporal offset between HBV-capturing shells and the addition of infectious HBV affects the neutralization capacity of the nanoshells, we infected cells with recombinant HBV (rHBV) expressing a secreted nanoluciferase. This allows more sensitive detection of HBV infection at a lower, more physiological MOI. rHBV was used at a MOI of 10 virions/cell, and nanoshells were added at a concentration of 15.9 nmol/L at the time of infection or at different time offsets of 1, 2, 3, 4, 6, 8, and 24 h before or after infection. To quantify infection, we measured luminescence in cell lysates 8 days post-infection as a readout. We found that pre-incubation of shells with HBV and simultaneous addition of both components led to comparable levels of neutralization (Figure 3E), indicating that the nanoshells readily engulf the infectious virus in the cell culture medium even at high dilution.

The relative timing of the addition of shells and viruses to cells impacted the neutralization capacity of the nanoshells. If shells were added prior to the virus, the degree of neutralization decreased with increasing temporal offset (Figure 3F). This was most likely due to progressive degradation of the shell material in the cell culture environment.^12^ On the other hand, administering shells *a posteriori* to exposing cells to viruses did not neutralize viruses for temporal offsets of 1 h or longer. This finding makes sense considering that the closed cell contact by HBV is expected to be established between 30 and 60 min.^29^ Once the viruses have entered the cells, they can no longer be sequestered by the HBV-capturing shells, because these shells are not expected to enter the cell.

## Conclusion

In previous work,^12^ we developed design principles and methods to construct DNA origami nanoshells and successfully trapped various viruses using different types of internal functionalization.^13,30^ In the present study, we show the first successful *in vitro* neutralization of a live human pathogen using these shells, whereas in previous work, non-infectious model particles, such as the HBV core and non-replicative viruses, were used. An additional distinctive feature is the achievement of neutralization enhancement of the efficacy of the recombinant IgG antibody MoMAb, which we used as a coating inside the shells to specifically trap HBV. TheIC_50_ decreased significantly from around 0.5 μmol/L^25^ per free anti-HBs IgG to ∼5 nmol/L when the IgG is mounted inside the shells. This corresponds to an IC_50_ of approximately 55 pmol/L of HBV-capturing shells. Consequently, our approach thus allows for a 2 orders of magnitude reduction in the required antibody quantity. The potency enhancements are presumably achieved by two mechanisms working in concert: multivalent binding of HBV virions to the anti-HBs antibody inside shells, which leads to avidity effects, and steric occlusion by the shell material, creating a physical barrier that prevents the virus from interacting with host cells. The latter was confirmed by a clear temporal offset when nanoshells were added at distinct time points before or after the virus. The medications available for HBV, particularly those that target the HBsAg, are often sidetracked by the substantial secretion of SVPs from infected cells.^20^ This is surely also the case for DNA nanoshells; however, as the TEM images suggest (Figure 2B), each nanoshell has the capacity to incorporate a substantial number of SVPs. This could potentially enhance their efficacy by reducing the number of SVPs in solution and allowing for actual hepatitis virions to be captured.

Our findings establish the shell system as a molecular framework for constructing new antivirals from components that have weak antiviral properties when used individually. Whether the nanoshell–virus complexes are taken up by antigen-presenting cells and induce a virus-specific T-cell response remains to be investigated. First results hint toward the uptake of the complexes by monocyte-derived dendritic cells (moDCs) (Figure S7 of the Supporting Information) but require further study. Another proposed advantage of our shells is the fact that they should be less prone to antibody-dependent infection enhancement, a mechanism where antibodies are not able to efficiently eliminate infected cells/viruses but where the antibody–virus complex is taken up by the cells; therefore, the virus can replicate within the cell. *In vivo* efficacy studies are crucial next steps to establish the therapeutic potential of the DNA shell system.

## Materials and Methods

### DNA Origami Shell Design, Self-Assembly, and Purification

The triangular shell subunits were designed and self-assembled as previously described^12^ using a scaffold with a length of 8064 bases.^31^ The resulting triangular monomers were purified using agarose gel purification with an agarose concentration of 1.5% and 0.5× Tris–borate–ethylenediaminetetraacetic acid (TBE) buffer containing 5.5 mM MgCl_2_. The leading bands were extracted from the gel, and the agarose was removed using the Corning Costar Spin-X centrifuge tube filters with a cellulose acetate membrane and a pore size of 0.45 μm. Residual agarose was removed by spinning the sample for 35 min at 21 000 rcf and collecting the supernatant. Details of the purification methods can be found in ref (32). The purified monomers were assembled to *T* = 1 nanoshells by increasing the MgCl_2_ concentration in the buffer to 40 mM and incubating at 40 °C for at least 8 days. The resulting *T* = 1 nanoshells are made up of 10 triangular subunits, each with nine single-stranded DNA sequences (“handles”) protruding into the cavity of the shell.

### Shell Stabilization

The *T* = 1 nanoshells were stabilized as described previously.^12^ This includes the strategy of UV point welding^26^ the individual subunit protrusions and recesses to ensure the structural integrity of the shell. The UV light source used is a custom built UV lamp with the deep ultraviolet light emission source (DUVLED) DUV310-SD353EN from Roither Laser Technik GmbH. The DUVLED emits light at a typical peak wavelength of 310 nm and is operated at an optical output power of 43 mW at 350 mA. For the irradiation procedure, the sample is placed into a self-made Teflon cuvette to maximize the reflection of the incoming UV light. All *T* = 1 nanoshell samples were irradiated 10 min with UV light prior to functionalization with antibodies. After functionalization, the construct was further stabilized by coating the structure using a 0.6:1 N/P ratio with a 1:1 mixture of K_10_–oligolysine (PL) and *K*_10_–PEG_5K_–oligolysine (PPL), a stabilization strategy described perviously^27^ and modified to stabilize higher order DNA origami structures.

### DNA Coupling of Anti-HBs Antibodies

The anti-S scFV-Linker-hIgG1Fcmut antibodies (MoMAb) used to capture subviral particles and HBV virions were previously described and characterized.^25^ Large-scale production was contracted to InVivo Biotech Services GmbH. The produced antibodies were modified with a 26 bp DNA sequence complementary to the handle sequence inside the nanoshells (Table 1).

**Table 1:** DNA Sequences for Antibody Attachment

function	sequence
sequence shell handles	5′-GCAGTAGAGTAGGTAGAGATTAGGCA-3′
sequence on antibody	5′-TGCCTAATCTCTACCTACTCTACTGC-thiol-3′

The modification was accomplished by connecting the thiol-modified DNA strand to the antibody via a sulfo-SMCC cross-linker from Thermo Scientific, using a ratio of antibody/DNA strand of 1:7 (Figures S2 and S3 of the Supporting Information). The resulting mixture was purified using the ion-exchange chromatography system proFire by Dynamic Biosensors.

### Nanoshells and HBV Subviral Particles and Virion Binding

The nanoshells were incubated with the antibodies in a handle/antibody ratio of 1:1 and incubated overnight at 25 °C. They were subsequently coated with the PL/PPL coating for at least 2 h. For TEM imaging, we added subviral particles in phosphate-buffered saline (PBS) buffer or purified hepatitis B viruses to the functionalized nanoshells and incubated for at least 4 h. For neutralization experiments, the functionalized nanoshell samples or the antibody-only samples were incubated with the hepatitis B virus sample at different ratios at 37 °C for 3 h.

### Negative Staining TEM

For TEM imaging, the samples were incubated on FCF-400-Cu TEM grids from Electron Microscopy Sciences with a Formvar carbon film, which was previously glow-discharged for 45 s with a charge of 35 mA. The incubation time ranged from 1 to 20 min depending upon the concentration of the sample. The samples were subsequently submerged in 2% aqueous uranyl formate solution containing 25 mM sodium hydroxide for staining and blotted dry with filter paper. Images were acquired using a FEI Tecnai T12 microscope at 120 kV and a Tietz TEMCAM-F416 camera, all operated with the software SerialEM. The magnification of the images ranged from 21000× to 52000×. The contrast of the images was subsequently globally enhanced with Fiji^33^ to show details of individual features.

### Neutralization Assays

To determine the neutralization capacity of the nanoshells, HepG2-NTCP cells were seeded in a collagenized 24 well plate at a density of 3 × 10^5^ cells/well in differentiation medium, which is Dulbecco’s modified Eagle’s medium (DMEM) high glucose supplemented with 10% fetal calf serum (FCS), 2.5% dimethyl sulfoxide (DMSO), penicillin/streptomycin, non-essential amino acids, l-glutamine, and sodium pyruvate (all Gibco Life Technologies) 3 days before infection. Different concentrations of nanoshells or MoMAb antibodies were mixed with purified HBV,^34^ genotype D, and incubated at 37 °C for 3 h. PEG was added to reach a final concentration of 4% (v/v) before adding the virus–nanoshell mixture to the cells at an MOI of 100 viral particles per cell. After 18 h, the inoculum was removed, cells were washed twice with PBS, and 1 mL of medium was added to the cells for further cultivation. At days 4 and 8 post-infection, cell culture medium was collected and HBeAg levels were determined by ELISA to calculate the neutralization capacity. In addition, cells were lysed 8 days post-infection to determine intracellular HBV cccDNA and rcDNA via qPCR.

To determine the prophylactic potential of the nanoshells, shells were added at different time points before infection of the HepG2-NTCP cells with a luciferase-expressing rHBV (genotype D, MOI of 10 viral particles/cell) in the presence of 4% PEG (v/v). To determine the therapeutic potential, nanoshells were added at different time points after infection. At 18 h after infection, the inoculum was removed, the cells were washed twice with PBS, and 1 mL of media was added to the cells. At days 4 and 8 post-infection, luciferase activity of 100 μL of cell culture medium was determined to calculate neutralization capacity.

## Supporting Information Available

Additional information on size comparisons, DNA–antibody coupling, overviews of negative stain electron microscopy (EM) data, dose–response curves, and uptake by dendritic cells (PDF)
