# Supplementary material for: Hepatitis B Virus Neutralization with DNA Origami Nanoshells
Source: ACS Appl Mater Interfaces. 2024 May 10;16(20):25836–42. doi: 10.1021/acsami.4c03700 (PMC11129107; doi:10.1021/acsami.4c03700)
Supplement: Supplementary file 1 — am4c03700_si_001.pdf [file am4c03700_si_001.pdf]

Supporting Information

Hepatitis B Virus Neutralization with DNA Origami Nanoshells

Elena M. Willner[1]‡, Fenna Kolbe[2]‡, Frank Momburg[3], Ulrike Protzer[2,4], Hendrik Dietz[1]**

[1] Department of Biosciences, School of Natural Sciences, Technical University of Munich, Garching near Munich, Germany & Munich Institute of Biomedical Engineering, Technical University of Munich, Boltzmannstraße 11, 85748 Garching, Germany.

[2] Institute of Virology, School of Medicine, Technical University of Munich, and Helmholtz Munich, Trogerstr. 30, 81675 Munich, Germany

[3] Translational Immunity Unit, German Cancer Research Center (DKFZ), Im Neuenheimer Feld, 69120 Heidelberg, Germany

[4] German Center for Infection Research (DZIF), Munich partner site, 81675 Munich, Germany

‡These authors contributed equally

* Please address correspondence to: protzer@tum.de, dietz@tum.de

Content

Figure S1: 3D rendering of the *HBV* and the T=1 nano-shell

Figure S2: DNA-antibody coupling with *Sulfo-SMCC*

Figure S3: Negative stain electron microscopy images

Figure S4: Agarose gel of the purified DNA-antibody product

Figure S5: Dose-response of *AB* only vs T=1 nano-shell neutralization

Figure S6: Statistically significant neutralization

Figure S7: Nanoshell uptake by dendritic cells

Table S1: Scaffold sequence.

Table S2: Staple sequences triangular monomer 1

Table S3: Staple sequences triangular monomer 2

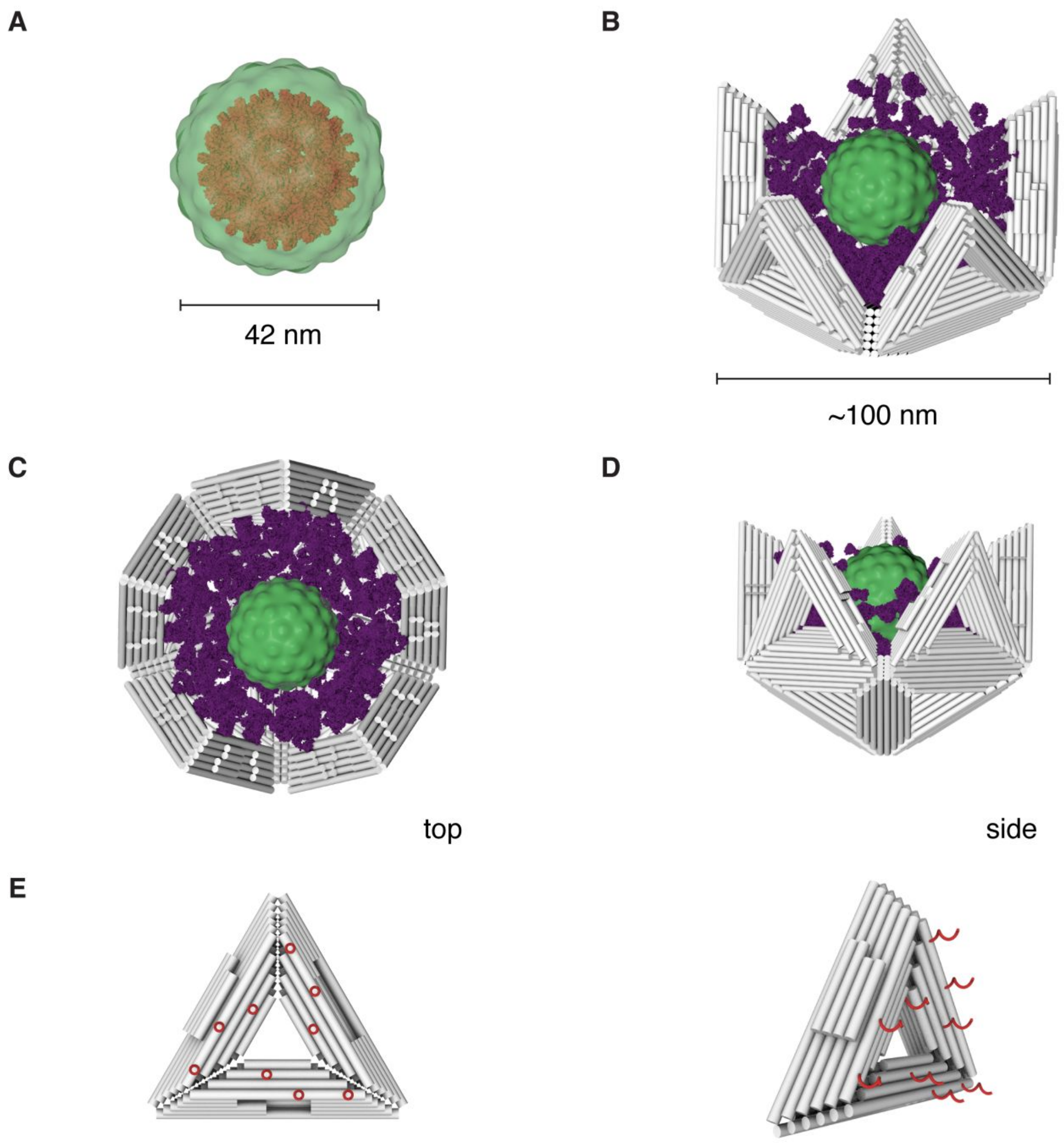

Figure S1. **3D rendering of the *HBV* and the T=1 nano-shell. (A)** 3D rendering of the Hepatitis B virus with its core particle colored in red and the envelope colored in green. (**B-D)** To scale rendering of the Hepatitis B virus embedded in the T=1 nano-shell. **(B)** View at an angle, **(C)** view from the top and **(D)** view from the side. **(E)** Illustration of the exact positions of the

protruding DNA handles, shown in red, used for antibody binding. Handles are not shown to scale.

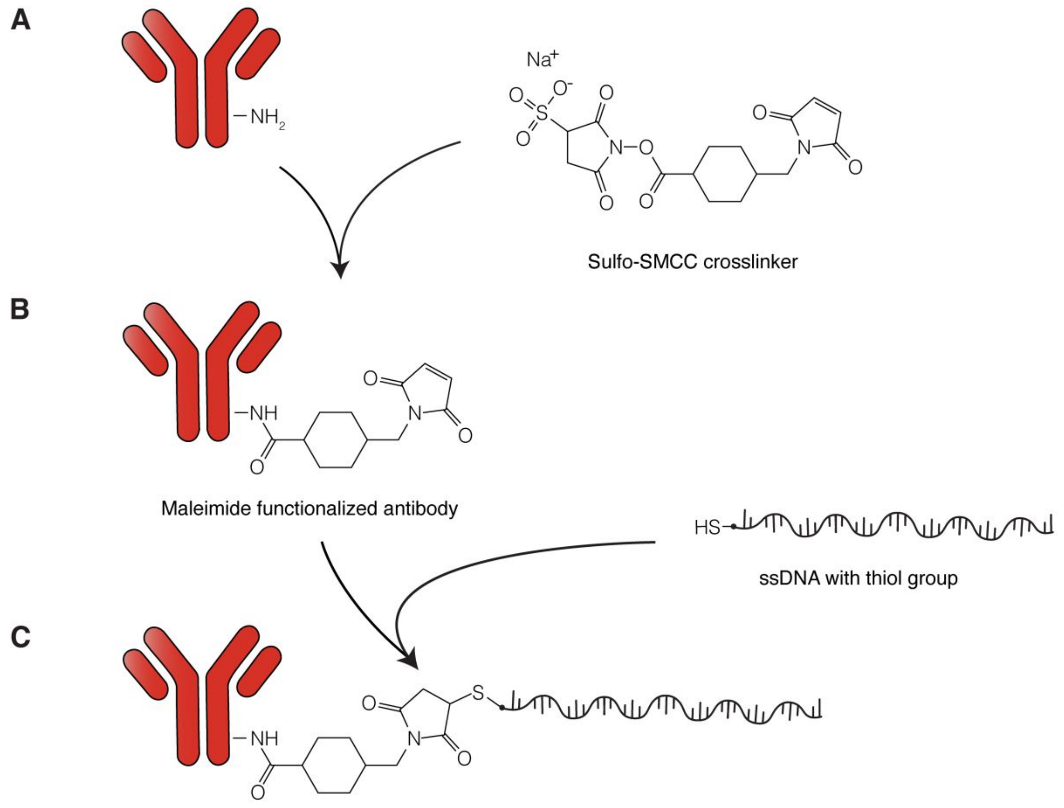

Figure S2. **Illustration of the DNA-antibody coupling with *Sulfo-SMCC*.**

(A) Antibody with free amine groups is mixed with a *Sulfo-SMCC* crosslinker. **(B)** Maleimide functionalized antibody is incubated with a thiolated single-stranded DNA handle. **(C)** Final DNA functionalized antibody product. Illustrated with information from [1].

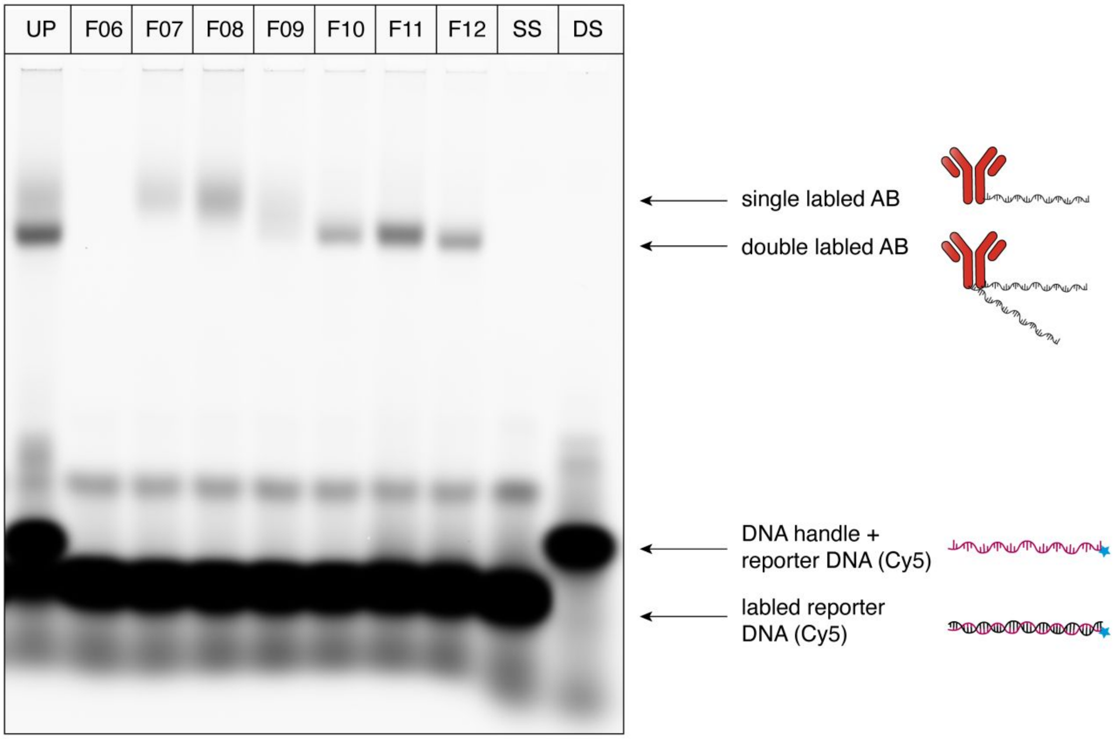

Figure S3. **Agarose gel of the purified DNA-antibody product.** 4% agarose gel analysis of the DNA-antibody product after purification with ion-exchange chromatography. *UP* stands for the unpurified product. *F06 – F12* stands for the purification fractions. *SS* is a single stranded DNA reporter with Cyanine 5 dye, complementary to the handle on the antibody. *DS* is the *SS* reporter with the complementary handle without the antibody.

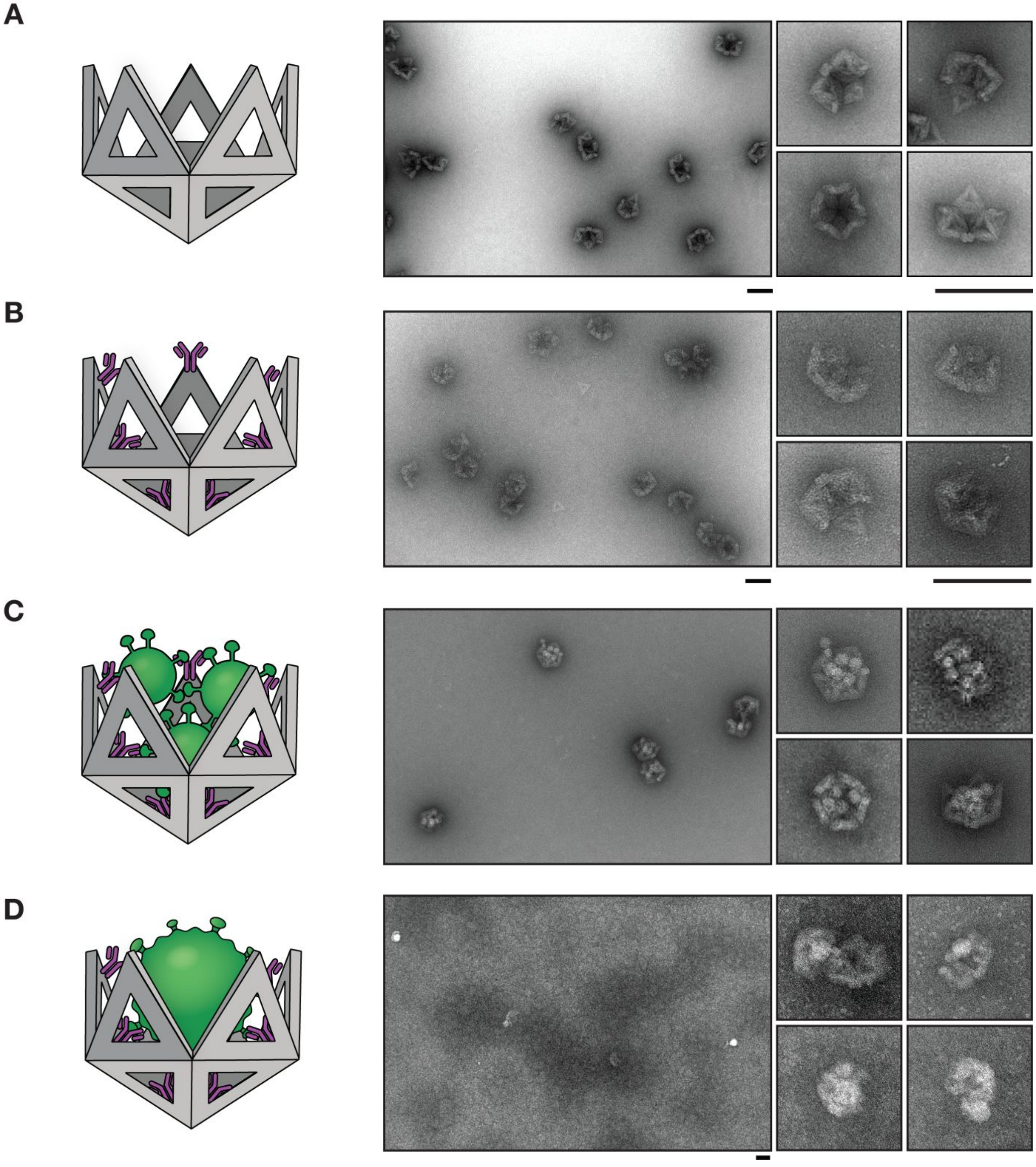

Figure S4. **Negative stain electron microscopy images.** Overview of the **(A)** DNA nano-shells, **(B)** antibody functionalized DNA nano-shells, **(C)** the capture of Hepatitis B subviral particles and **(D)** the capture of *HBV* viruses fixed with formaldehyde. Scale bar indicates 100 nm.

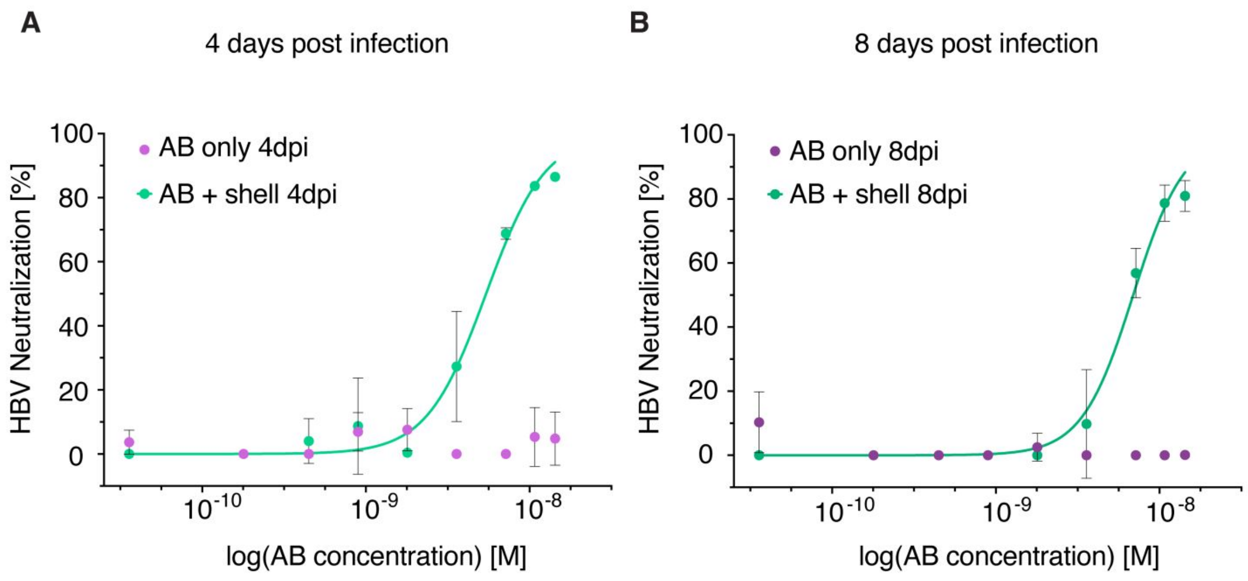

Figure S5. **Dose-response of *AB* only vs T=1 nano-shell neutralization**. *IC50* curves of the *HBV* neutralization data using functionalized nano-shells using the *HBeAg* detection. **(A)** Shows the neutralization capacity 4 days after the *HBV* infection (*4dpi*) and **(B)** 8 days after infection (*8dpi*). Neutralization data was fitted using a nonlinear regression and resulted in IC50 values of 5.4 nM ± 1.3 nM and 6.9 nM ± 1.2 nM for *4dpi* and *8dpi* respectively. The data is represented as mean + s.d. and is composed of n=3 biologically independent experiments.

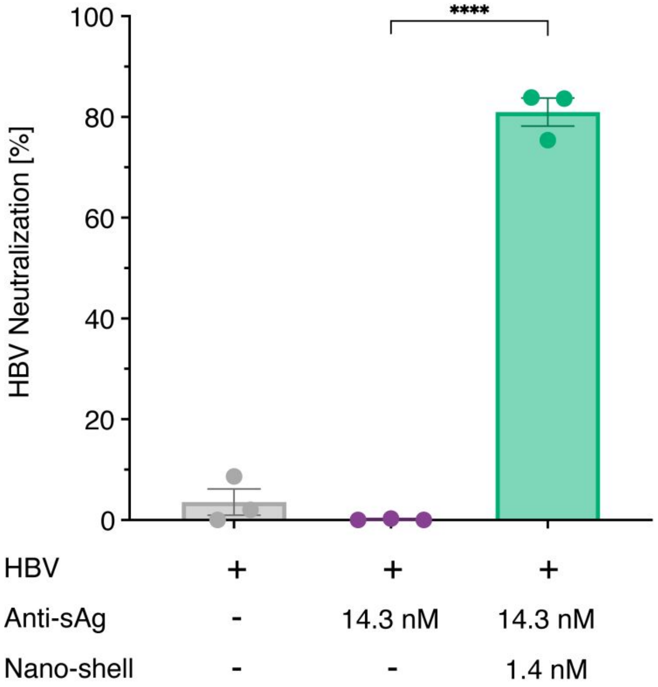

Figure S6. **Statistically significant neutralization.** One-way analysis of variance (*ANOVA*) of the highest tested nano-shell concentration to study statistically significant virus blocking compared with the antibody only sample. The functionalized nano-shells show a statistically significant higher neutralization capacity than the sample using the antibodies only (**** $P < 0.0001$).

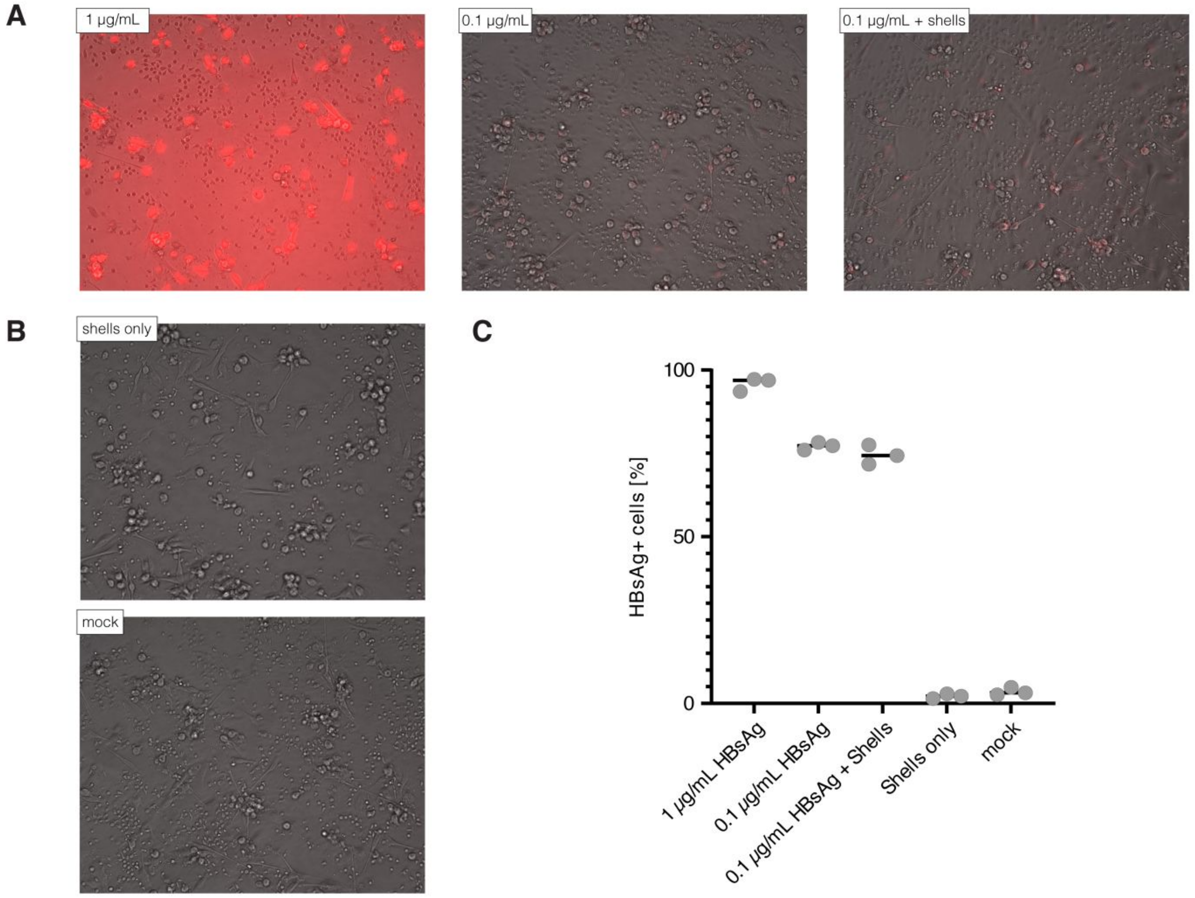

Figure S7. Nano-shell uptake by dendritic cells. (A) Nano-shells with fluorescently labeled *HBsAg* at different concentrations added to dendritic cells (*moDC*) **(B)** Cell control with shells only and mock. **(C)** Data on the amount of internalized HBsAg into the *moDC* cell line using n = 3 biological independent measurements.

Table S1 | Scaffold sequence.

M13MP18 modified sequence with length of 8064 bases.[2]
GGCAATGACCTGATAGCCTTTGTAGATCTCTCAAAAATAGCTACCCTCTCCGGCAT<br>TAATTTATCAGCTAGAACGGTTGAATATCATATTGATGGTGATTTGACTGTCTCCG<br>GCCTTTCTCACCCTTTTGAATCTTTACCTACACATTACTCAGGCATTGCATTTAAAA<br>TATATGAGGGTTCTAAAAATTTTTATCCTTGCGTTGAAATAAAGGCTTCTCCCGCA<br>AAAGTATTACAGGGTCATAATGTTTTTGGTACAACCGATTTAGCTTTATGCTCTGA<br>GGCTTTATTGCTTAATTTTGCTAATTCTTTGCCTTGCCTGTATGATTTATTGGATGT<br>TAATGCTACTACTATTAGTAGAATTGATGCCACCTTTTCAGCTCGCGCCCCAAATG<br>AAAATATAGCTAAACAGGTTATTGACCATTTGCGAAATGTATCTAATGGTCAAAC<br>TAAATCTACTCGTTCGCAGAATTGGGAATCAACTGTTATATGGAATGAAACTTCCA<br>GACACCGTACTTTAGTTGCATATTTAAAACATGTTGAGCTACAGCATTATATTCAG<br>CAATTAAGCTCTAAGCCATCCGCAAAAATGACCTCTTATCAAAAGGAGCAATTAA<br>AGGTACTCTCTAATCCTGACCTGTTGGAGTTTGCTTCCGGTCTGGTTCGCTTTGAA<br>GCTCGAATTAAAACGCGATATTTGAAGTCTTTCGGGCTTCCTCTTAATCTTTTTGAT<br>GCAATCCGCTTTGCTTCTGACTATAATAGTCAGGGTAAAGACCTGATTTTTGATTT<br>ATGGTCATTCTCGTTTTCTGAACTGTTTAAAGCATTTGAGGGGGATTCAATGAATA<br>TTTATGACGATTCCGCAGTATTGGACGCTATCCAGTCTAAACATTTTACTATTACC<br>CCCTCTGGCAAAACTTCTTTTGCAAAAGCCTCTCGCTATTTTGGTTTTTATCGTCGT<br>CTGGTAAACGAGGGTTATGATAGTGTTGCTCTTACTATGCCTCGTAATTCCTTTTG<br>GCGTTATGTATCTGCATTAGTTGAATGTGGTATTCCTAAATCTCAACTGATGAATC<br>TTTCTACCTGTAATAATGTTGTTCCGTTAGTTCGTTTTATTAACGTAGATTTTTCTTC<br>CCAACGTCCTGACTGGTATAATGAGCCAGTTCTTAAAATCGCATAAGGTAATTCAC<br>AATGATTAAAGTTGAAATTAAACCATCTCAAGCCCAATTTACTACTCGTTCTGGTG<br>TTTCTCGTCAGGGCAAGCCTTATTCACTGAATGAGCAGCTTTGTTACGTTGATTTG

GGTAATGAATATCCGGTTCTTGTCAGATTACTCTTGATGAAGGTCAGCCAGCCTAT
GCGCCTGGTCTGTACACCGTTCACTGTCCTCTTTCAAAGTTGGTCAGTTCGGTTCCC
TTATGATTGACCGTCTGCGCCTCGTTCCGGCTAAGTAACATGGAGCAGGTCGCGG
ATTTCGACACAATTTATCAGGCGATGATACAAATCTCCGTTGTACTTTGTTTCGCG
CTTGGTATAATCGCTGGGGGTCAAAGATGAGTGTTTTAGTGTATTCTTTTGCCTCTT
TCGTTTTAGGTTGGTGCCTTCGTAGTGGCATTACGTATTTTACCCGTTTAATGGAA
ACTTCCTCATGAAAAAGTCTTTAGTCCTCAAAGCCTCTGTAGCCGTTGCTACCCTC
GTTCCGATGCTGTCTTTCGCTGCTGAGGGTGACGATCCCGCAAAAGCGGCCTTTAA
CTCCCTGCAAGCCTCAGCGACCGAATATATCGGTTATGCGTGGGCGATGGTTGTTG
TCATTGTGGCGCAACTATCGGTATCAAGCTGTTTAAGAAATTCACCTCGAAAGCA
AGCTGATAAACCGATACAATTAAAGGCTCCTTTTGGAGCCTTTTTTTTGGAGATTT
TCAACGTGAAAAAATTATTATTCGCAATTCCTTTAGTTGTTCCTTTCTATTCTCACT
CCGCTGAAACTGTTGAAAGTTGTTTAGCAAAATCCCATACAGAAAATTCATTTACT
AACGTCTGGAAAGACGACAAAACTTTAGATCGTTACGCTAACTATGAGGGCTGTC
TGTGGAATGCTACAGGCGTTGTAGTTTGTACTGGTGACGAAACTCAGTGTTACGGT
ACATGGGTTCCTATTGGGCTTGCTATCCCTGAAAATGAGGGTGGTGGCTCTGAGG
GTGGCGGTTCTGAGGGTGGCGGTTCTGAGGGTGGCGGTACTAAACCTCCTGAGTA
CGGTGATACACCTATTCCGGGCTATACTTATATCAACCCTCTCGACGGCACTTATC
CGCCTGGTACTGAGCAAAACCCCGCTAATCCTAATCCTTCTCTTGAGGAGTCTCAG
CCTCTTAATACTTTCATGTTTCAGAATAATAGGTTCCGAAATAGGCAGGGGGCATT
AACTGTTTATACGGGCACTGTTACTCAAGGCACTGACCCCGTTAAAACTTATTACC
AGTACACTCCTGTATCATCAAAAGCCATGTATGACGCTTACTGGAACGGTAAATTC
AGAGACTGCGCTTTCCATTCTGGCTTTAATGAGGATTTATTTGTTTGTGAATATCA
AGGCCAATCGTCTGACCTGCCTCAACCTCCTGTCAATGCTGGCGGCGGCTCTGGTG
GTGGTTCTGGTGGCGGCTCTGAGGGTGGTGGCTCTGAGGGTGGCGGTTCTGAGGG
TGGCGGCTCTGAGGGAGGCGGTTCCGGTGGTGGCTCTGGTTCCGGTGATTTTGATT
ATGAAAAGATGGCAAACGCTAATAAGGGGGCTATGACCGAAAATGCCGATGAAA
ACGCGCTACAGTCTGACGCTAAAGGCAAACTTGATTCTGTCGCTACTGATTACGGT
GCTGCTATCGATGGTTTCATTGGTGAGTTTCCGGCCTTGCTAATGGTAATGGTGCT
ACTGGTGATTTTGCTGGCTCTAATTCCCAAATGGCTCAAGTCGGTGACGGTGATAA
TTCACCTTTAATGAATAATTTCCGTCAATATTTACCTTCCCTCCCTCAATCGGTTGA
ATGTCGCCCTTTTGTCTTTGGCGCTGGTAAACCATATGAATTTTCTATTGATTGTGA

CAAAATAAACTTATTCCGTGTGTCTTTGCGTTTCTTTTATATGTTGCCACCTTTATG
TATGTATTTTCTACGTTTGCTAACATACTGCGTAATAAGGAGTCTTAATCATGCCA
GTTCTTTTGGGTATTCCGTTATTATTGCGTTTCCTCGGTTTCCTTCTGGTAACTTTGT
TCGGCTATCTGCTTATTTTCTTAAAAAGGGCTTCGGTAAGATAGCTATTGCTATTTC
ATTGTTTCTTGCTCTTATTATTGGGCTTAACTCAATTCTTGTGGGTTATCTCTCTGA
TATTAGCGCTCAATACCCTCTGACTTTGTTCAGGGTGTTCAGTTAATTCTCCCGTCT
AATGCGCTTCCCTGTTTTTATGTTATTCTCTCTGTAAAGGCTGCTATTTTCATTTTTG
ACGTTAAACAAAAAATCGTTTCTTATTTGGATTGGGATAAATAATATGGCTGTTTA
TTTTGTAACTGGCAAATTAGGCTCTGGAAAGACGCTCGTTAGCGTTGGTAAGATTC
AGGATAAAATTGTAGCTGGGTGCAAAATAGCAACTAATCTTGATTTAAGGCTTCA
AAACCTCCCGCAAGTCGGGAGGTTCGCTAAAACGCCTCGCGTTCTTAGAATACCG
GATAAGCCTTCTATATCTGATTTGCTTGCTATTGGGCGCGGTAATGATTCCTACGA
TGAAAATAAAAACGGCTTGCTTGTTCTCGATGAGTGCGGTACTTGGTTTAATACCC
GTTCTTGGAATGATAAGGAAAGACAGCCGATTATTGATTGGTTTCTACATGCTCGT
AATTAGGATGGGATATTATTTTTCTTGTTCAGGACTTATCTATTGTTGATAAACAG
GCGCGTTCTGCATTAGCTGAACATGTTGTTTATTGTCGTCGTCTGGACAGAATTAC
TTTACCTTTTGTCGGTACTTTATATTCTCTTATTACTGGCTCGAAAATGCCTCTGCC
TAAATTACATGTTGGCGTTGTTAAATATGGCGATTCTCAATTAAGCCCTACTGTTG
AGCGTTGGCTTTATACTGGTAAGAATTTGTATAACGCATATGATACTAAAAGGCTT
TTTCTAGTAATTATGATTCCGGTGTTTATTCTTATTTAACGCCTTATTTATCACACG
GTCGGTATTTCAAACCATTAAATTTAGGTCAGAAGATGAAATTAACTAAAATATA
TTTGAAAAAGTTTTCTCGCGTTCTTTGTCTTGCGATTGGATTTGCATCGCATTTACA
TATAGTTATATAACCCAACCTAAGCCGGAGGTTAAAAAGGTAGTCTCTCAGACCT
ATGATTTTGATAAATTCACTATTGACTCTTCTCAGCGTCTTAATCTAAGCTATCGCT
ATGTTTTCAAGGATTCTAAGGGAAAATTAATTAATAGCGACGATTTACAGAAGCA
AGGTTATTCACTCACATATATTGATTTATGTACTGTTTCCATTAAAAAAGGTAATT
CAAATGAAATTGTTAAATGTAATTAATTTTGTTTTCTTGATGTTTGTTTCATCATCT
TCTTTTGCTCAGGTAATTGAAATGAATAATTCGCCTCTGCGCGATTTTGTAACTTG
GTATTCAAAGCAATCAGGCGAATCCGTTATTGTTTCTCCCGATGTAAAAGGTACTG
TTACTGTATATTCATCTGACGTTAAACCTGAAAATCTACGCAATTTCTTTATTTCTG
TTTTACGTGCAAATAATTTTGATATGGTAGGTTCTAACCCTTCCATTATTCAGAAG
TATAATCCAAACAATCAGGATTATATTGATGAATTGCCATCATCTGATAATCAGGA

ATATGATGATAATTCCGCTCCTTCTGGTGGTTTCTTTGTTCCGCAAAATGATAATGT
TACTCAAACTTTTAAAATTAATAACGTTCGGGCAAAGGATTTAATACGAGTTGTCG
AATTGTTTGTAAAGTCTAATACTTCTAAATCCTCAAATGTATTATCTATTGACGGC
TCTAATCTATTAGTTGTTAGTGCTCCTAAAGATATTTTAGATAACCTTCCTCAATTC
CTTTCAACTGTTGATTTGCCAACTGACCAGATATTGATTGAGGGTTTGATATTTGA
GGTTCAGCAAGGTGATGCTTTAGATTTTTCATTTGCTGCTGGCTCTCAGCGTGGCA
CTGTTGCAGGCGGTGTTAATACTGACCGCCTCACCTCTGTTTTATCTTCTGCTGGTG
GTTCGTTCGGTATTTTTAATGGCGATGTTTTAGGGCTATCAGTTCGCGCATTAAAG
ACTAATAGCCATTCAAAAATATTGTCTGTGCCACGTATTCTTACGCTTTCAGGTCA
GAAGGGTTCTATCTCTGTTGGCCAGAATGTCCCTTTTATTACTGGTCGTGTGACTG
GTGAATCTGCCAATGTAAATAATCCATTTCAGACGATTGAGCGTCAAAATGTAGG
TATTTCCATGAGCGTTTTTCCTGTTGCAATGGCTGGCGGTAATATTGTTCTGGATAT
TACCAGCAAGGCCGATAGTTTGAGTTCTTCTACTCAGGCAAGTGATGTTATTACTA
ATCAAAGAAGTATTGCTACAACGGTTAATTTGCGTGATGGACAGACTCTTTTACTC
GGTGGCCTCACTGATTATAAAAACACTTCTCAGGATTCTGGCGTACCGTTCCTGTC
TAAAATCCCTTTAATCGGCCTCCTGTTTAGCTCCCGCTCTGATTCTAACGAGGAAA
GCACGTTATACGTGCTCGTCAAAGCAACCATAGTACGCGCCCTGTAGCGGCGCAT
TAAGCGCGGCGGGTGTGGTGGTTACGCGCAGCGTGACCGCTACACTTGCCAGCGC
CCTAGCGCCCGCTCCTTTCGCTTTCTTCCCTTCCTTTCTCGCCACGTTCGCCGGCTT
TCCCCGCAAGCTCTAAATCGGGGGCTCCCTTTAGGGTTCCGATTTAGTGCTTTACG
GCACCTCGACCCCAAAAAACTTGATTTGGGTGATGGTTCACGTAGTGGGCCATCG
CCCTGATAGACGGTTTTTCGCCCTTTGACGTTGGAGTCCACGTTCTTTAATAGTGG
ACTCTTGTTCCAAACTGGAACAACACTCAACCCTATCTCGGGCTATTCTTTTGATTT
ATAAGGGATTTTGCCGATTTCGGAACCACCATCAAACAGGATTTTCGCCTGCTGGG
GCAAACCAGCGTGGACCGCTTGCTGCAACTCTCTCAGGGCCAGGCGGTGAAGGGC
AATCAGCTGTTGCCCGTCTCACTGGTGAAAAGAAAAACCACCCTGGCGCCCAATA
CGCAAACCGCCTCTCCCCGCGCGTTGGCCGATTCATTAATGCAGCTGGCACGACA
GGTTTCCCGACTGGAAAGCGGGCAGTGAGCGCAACGCAATTAATGTGAGTTAGCT
CACTCATTAGGCACCCCAGGCTTTACACTTTATGCTTCCGGCTCGTATGTTGTGTG
GAATTGTGAGCGGATAACAATTTCACACAGGAAACAGCTATGACCATGATTACGA
ATTCGAGCTCGGTACCCGGGGATCCTCAACTGTGAGGAGGCTCACGGACGCGAAG
AACAGGCACGCGTGCTGGCAGAAACCCCCGGTATGACCGTGAAAACGGCCCGCC

GCATTCTGGCCGCAGCACCACAGAGTGCACAGGCGCGCAGTGACACTGCGCTGGA
TCGTCTGATGCAGGGGGCACCGGCACGCTGGCTGCAGGTAACCCGGCATCTGATG
CCGTTAACGATTTGCTGAACACACCAGTGTAAGGGATGTTTATGACGAGCAAAGA
AACCTTTACCCATTACCAGCCGCGGGCAACAGTGACCCGGCTCATACCGCAACCG
CGCCCGGCGGATTGAGTGCGAAAGCGCCTGCAATGACCCCGCTGATGCTGGACAC
CTCCAGCCGTAAGCTGGTTGCGTGGGATGGCACCACCGACGGTGCTGCCGTTGGC
ATTCTTGCGGTTGCTGCTGACCAGACCAGCACCACGCTGACGTTCTACAAGTCCGG
CACGTTCCGTTATGAGGATGTGCTCTGGCCGGAGGCTGCCAGCGACGAGACGAAA
AAACGGACCGCGTTTGCCGGAACGGCAATCAGCATCGTTTAACTTTACCCTTCATC
ACTAAAGGCCGCCTGTGCGGCTTTTTTTACGGGATTTTTTTATGTCGATGTACACA
ACCGCCCAACTGCTGGCGGCAAATGAGCAGAAATTTAAGTTTGATCCGCTGTTTCT
GCGTCTCTTTTTCCGTGAGAGCTATCCCTTCACCACGGAGAAAGTCTATCTCTCAC
AAATTCCGGGACTGGTAAACATGGCGCTGTACGTTTCGCCGATTGTTTCCGGTGGG
TTATCCGTTCCCGTGGCGGCTCCACCTCTGAAAGCTTGGCACTGGCCGTCGTTTTA
CAACGTCGTGACTGGGAAAACCCTGGCGTTACCCAACTTAATCGCCTTGCAGCAC
ATCCCCCTTTCGCCAGCTGGCGTAATAGCGAAGAGGCCCGCACCGATCGCCCTTC
CCAACAGTTGCGCAGCCTGAATGGCGAATGGCGCTTTGCCTGGTTTCCGGCACCA
GAAGCGGTGCCGGAAAGCTGGCTGGAGTGCGATCTTCCTGAGGCCGATACTGTCG
TCGTCCCCTCAAACTGGCAGATGCACGGTTACGATGCGCCCATCTACACCAACGT
GACCTATCCCATTACGGTCAATCCGCCGTTTGTTCCCACGGAGAATCCGACGGGTT
GTTACTCGCTCACATTTAATGTTGATGAAAGCTGGCTACAGGAAGGCCAGACGCG
AATTATTTTTGATGGCGTTCCTATTGGTTAAAAAATGAGCTGATTTAACAAAAATT
TAATGCGAATTTTAACAAAATATTAACGTTTACAATTTAAATATTTGCTTATACAA
TCTTCCTGTTTTTGGGGCTTTTCTGATTATCAACCGGGGTACATATGATTGACATGC
TAGTTTTACGATTACCGTTCATCGATTCTCTTGTTTGCTCCAGACTCTCA

Table S2 | Staple sequences triangular monomer 1.

Triangular monomer making up the pentameric base of the DNA origami nanoshell.[3]

Name	Sequence
side1_core_1	AGGGGACGACGACAGTTTTTATCGGCCTCAGGAAACCGTGCATC
side1_core_2	CCGAACAAGAATACCCAAAAGAACCATACATAACAGCCATGTTTTGAA
side1_core_3	TTAGACGGGAGAACCCGAAGCCCTTCCTTATTTGCAGCCA
side1_core_4	GAATCGATTCTACTAAGCTATATTTTCATTTAAGATTGATCAGAA
side1_core_5	CCAATAATAAGAGCAAGAGCAGATAGAAACAGGGAACGTCAA
side1_core_6	GTGTACAGACCAGGCGCATAGGCTATGCCACTGAGGCGCAAAACAGCT
side1_core_7	ACTATTATCTGGAGCACAAACTAATAGCGCGAAACAAAGTGCTCCAT
side1_core_8	AGAAAAATCTTTCATCAAGAGTAATCTTGACATTTTTGAACCGGATATTCAT
side1_core_9	AAGCAAACTTAATTGCGTCTGGAAATATTTTAATTTTTTGCAATGC
side1_core_10	CTTAGAGCTCCAACAGGTCAGGATTTTTTTGAGAGTAC
side1_core_11	CTAGCATGCAGCAAGCCCAATAGCGAACGATCTAAAGTT
side1_core_12	AGATTTGTAACACTCATAGTTAGCGTAATTATGAAACA
side1_core_13	AATATTTGCATTAAATTGTTTAGACTGTTTTTATAGGCATCGTA
side1_core_14	TCACGACGTTGAGCGCTAATATCCGCAAGTCAGATTGAATA
side1_core_15	AGAGCCACCACCCTCAACCCTCAGATAGCTATAGCTAGCAAGGACCAT
side1_core_16	AAGCCCCATTTTCAGGGATACCGTCGCCATTCAGGCTGCGCAACTG
side1_core_17	TATGTTAGCCGGAGACAGTCAAATCACCATCACGCGAGTCGAAAT
side1_core_18	TGATACCGCAACCTTTAATTGTATCGGTTTTTTTATCAGCTCAC
side1_core_19	TTCTAAGAAGAGGACAAGAGGCAACCGCGACCTACAACGGGGCTATCA
side1_core_20	TGATAAGAGGTCTTTTTTTTTTGCGGATGGTCATTATA

side1_core_21	CCTAATTTCAACGCTCGGGTTATATAACTATACTGTAAATAGAGAGAA
side1_core_22	CATCAATGAACGGTAATCGACCATGTACCGTATCATCG
side1_core_23	GCACCATTACCATTGAAAAGGTGGATTAAGCAACGGAGATCTACAAA
side1_core_24	ACCAGTAAAAGTAAAACAATGCTGAACACACCCTCGGCGATC
side1_core_25	GGTGCGGGCCTCTTCGTGATTGCTGGGTAATTTAACATAA
side1_core_26	AATGGGAGGGATTTTGCTTTTTTAACAACTTTCAAATTTTTTC
side1_core_27	ATCATACCCTGAGAGTGATAAATGTTACTTAGGAACCGA
side1_core_28	AATTTCTTAAAACGAACTAATTTTTGGAACAACATTATCCAGTCAG
side1_core_29	AGGCAAGGGATAAAAATTTTTAGAACCTTTTTTCATGTTTCATT
side1_core_30	CAATAACGGATTCGCCCTATTACGCCAGCTGGCGAAAGG
side1_core_31	CGCAGAGGGAATTAACTGAACACCAAATAGCAAACCGCCA
side1_core_32	TTTCCAGATCCAGCCATCACCAGTAAACAAGAGGTCATTG
side1_core_33	TTCTGTATTAGGTCACGTTGGTGTAGATGGGCCCAGGCAA
side1_core_34	ACGTTGAATTTTTATCTCCAAAAAAAAAGCAACCATCGCCCACGC
side1_core_35	AGCTATTTTTGACAGAAATTGTGGCGTTTTATCCGGTA
side1_core_36	TAATGCCGATTCAACCTGTGTAGGTAAAGATTCAAAAGGTTATTTC
side1_core_37	CTTTAATTTAGTCAGAAGCTTTTTAAGCGGATTGCATACCCTG
side1_core_38	GCCGGAAACGTACCCCGGTTGATATATAAGCA
side1_core_39	TTTGGGAATTAGAGCCAGCAAGCCGCCACC
side1_core_40	GTGAATAACCTTGCTTTGTAAATGAAATGAAACAAAATAAAAGGTGGC
side1_core_41	GGGAGGGAAGGTATTATCACGAAAATATGGCATG
side1_core_42	GCCAGTTAATAGCAGCCTTAGACGCTGAGAAG
side1_core_43	GCTTTCCGGTCAATCATATGTCCAATACTGCGGA

side1_core_44	CGTCACCGACTTGGGGTCGGTTGTACCAAAAACAT
side1_core_45	GATCGCACCGTTAGTACTGTAGCATTCCACAGGCGATTAT
side1_core_46	CTTAGGTTAACAGTAGGGCTTAATAGCCGTTTCCAGCTACAA
side1_core_47	CTGATGCATTAAAATTCTTACCAGCCGCGCCCGCCTTAAATCAAGATT
side1_core_48	CAAAGAACCCTTAAGAAACGATTTATTAAGACTTTTAAGA
side1_core_49	AAGGTGAAAATATTGAAAAGACAAAAGGGCGGCGGGAG
side1_core_50	ACCAAGTTACCAACCTAAAACGAAGATGAACGACTGACCAACTTTGAA
side1_core_51	CATGAGGAAGTTTTTTTCCATTAAATAATTTTTCGATATATTCGGT
side1_core_52	GAGGTTTAGTACCGCCAAAACAGGGGGGCGCGCTTTAAAA
side1_core_53	AATATGATGAGAGGGTAACGCAAGCAAAGAATTAGCAAA
side1_core_54	AATAGCAAGCAAATCAGATATAGTCAAATATATCCCAATCCAAAGAT
side1_core_55	CTGAGTAAGTTCTAGCGCTCCTTTATCATAAGGCCGGAAC
side1_core_56	CCGGAATCATAATTACATTTAATGAAACTTTT
side1_core_57	GCATAAAGCTAAAAGAAGCCTGTGAGAAAGGCCGATTGA
side1_core_58	ACGAAGGCGCGCCGACAATGACAAGCTCCAAAAGGAGCGCAATGAATT
side1_core_59	ACGGGTAAAATACGTAGGCTGACCACGTTAATGACGGTCA
side1_core_60	TTATTTTCATCGTAGGAATCATTATATAAAGC
side1_core_61	TATGACCCTGTAATACTTTATAAAGCCTCAGA
side1_core_62	AAGGCTTAGCGAACCTCCCGACTTACATTCAACAAACGTA
side1_core_63	GGAGGTCAATAACCTTTTTTTTATAGTAGTAGTTTTATTAATAGAT
side1_core_64	ATTTCATTTGAATTACCTTTTTTAAGAAGATGATTCATTT
side2_core_1	CCGCCACCCCTCATTAAAGCCAGAATGTTTTTAAAGATTCATTA
side2_core_2	ATCGGCTGTCTTTCCTTATCATTCTTAGGCAGTAAGTCCTGTGAATTT

side2_core_3	ATGGTTTAAACATATAAAAGAAACGTTTTTAAAGACAC
side2_core_4	CCAGCGCCCGGAAATTCGCAGTCTCTTTTTGAATTTACCGTTCCAG
side2_core_5	AGGCGGTTAGAAACCAATCAATACTAATTTACGAGCATG
side2_core_6	CTCAGAATTAAGAGGGCCCGTATGTTTATCAATCCCATC
side2_core_7	TTTTCCCTTTTTTAGAATCCTTGAAAACCATAGGTCTGAGAGAC
side2_core_8	GTGCCAGCTGCATTAATGAATCGGCCAACGCCAGGGTGGTTTTTCTT
side2_core_9	TTTTATCCTGGGTATTAAACCAAGTACCGCACTTTTTCATCGAGAACAAGCA
side2_core_10	GGGGTCAGAATGCCCCAAATAAATCTCAGAGCCACCACC
side2_core_11	GACTGTAGCGCGTACCGGAACCTCAGAGCGGGGAACCTATTATT
side2_core_12	TGAGGCCAGTTGCTTTGTAATAACATCACGCCCCGCCAGCATTGACAGGAG
side2_core_13	GTTAGAACTCAAACTACCTGAAAGC
side2_core_14	TCACTGCCCGCTTTCCAGTCGGGAGTTAACGG
side2_core_15	CTGTTTGACATCAGATGTCATAAACATCCCTTAGCACCGTTTAAAGAA
side2_core_16	ACCACCAGAGCCGTTGATATTTGATACAGGAGTGAGTAAA
side2_core_17	TAACTGATTGTTTGGATTTCAGGGCGATGGCAGCTTGACAGCGGAAT
side2_core_18	TGGTGGTTACAAGAGTCCACAATCCGCCGGGC
side2_core_19	AAAAGAATTAAGTACTATGCCGTACTGGTAATAAGTTTTAACTTGCGTA
side2_core_20	TCACGCAATGTTTTTATAATCAGTTCACCACCCTTATAAATC
side2_core_21	AGAGTCTGTCCATTGATTAGACGAGCGCCGCGC
side2_core_22	TGAGAATCGCTTTTTATATTTAACTACCTTTTTTTTTAACCTCCGG
side2_core_23	AACGCCAACATGTAATCAAGAACGAATCTTACGAACAAGA
side2_core_24	TTCAGCTAATGCAGAAAGTAATTCTGAAACAGAAGGATTACCACCGG
side2_core_25	CACGGAATAACCGAGGAAATTTTTGCAATAATAACGAGTTACC

side2_core_26	AAACAGTTTGCCTTGATAAGCGTCATACATGGCTTTTGACACAAAC
side2_core_27	GACGACGACAAAGCCCGAGATAGGTTAATGCGGAGAAAGG
side2_core_28	CTTTGCTCGCCGGGTTACCTGCAGCGTTGCGCCTGAGAGAGT
side2_core_29	CTGCCTATTTCGCGCACAACATGTTGGGCGCGCGGGGAG
side2_core_30	GTTGAGTAAAGGGCGAAAAACCGTCTAATAAACGTGGC
side2_core_31	ATCAATATAATCCCTATGTTTACCAGTCCCGGAATTTGTGAGAGAT
side2_core_32	AAGGAGCGCGTAACCACCACACCCACGTATAAGGCAAAATGTGAGACG
side2_core_33	AGTCAATACAACGCTAACGATTTTTCGTCTTTCCAGAGTTTTGCAC
side2_core_34	GATGAATATACAGTAAGCTGCAAGGCGATTAAGTTGGGTACGAAACGT
side2_core_35	CTGGTCTGGTCAGCAGTAGCTCTCACGGAAAAAGAGACG
side2_core_36	CCGCTACATTGTTGCCTGAGTAGAGAGGCAGGGCATTTTCGGT
side2_core_37	GGGATGTCAGTACCTTTTTTTTTCATCGGGAGAAATTAATTAA
side2_core_38	CTGTCCATAATGGAAGGGTTAGGGAACGGAACCAGGCGGATAAAATTGAGTTAAGC
side2_core_39	CCTTGCTAAGGGAAGATTTAGCCACTACTGATTATAGACTTT
side2_core_40	GGTCATAATCAAAATCTTTCATCGTCAGACGACGCCACCAGAACC
side2_core_41	CAGAGCCAGGATTAGCGGGGCGCTTCTGAA
side2_core_42	TCTTTTAATAGCCCCCTTATTAGCGGCCTTTAGCGTCA
side2_core_43	ACAGCGCCCACCGGAAACAATCGGAAGGTGCCGTCGAGAGTATCACCG
side2_core_44	CTCCGTGGTGAAGGGACAACCGCATCACCCAACGTGGACT
side2_core_45	TTTGTCACAATCTTTTTATAGAAAATTCATAGTTGCTA
side2_core_46	CAGATGATGGCAATTCCCAGAAGGGGGGAAAGTTTGCCA
side2_core_47	CGGAACAAAGAAACCAAGCCCGGATCAAGTTTCCGGTTTTGCTCAGT
side2_core_48	TCAGTAGCGACAGAAATAGGTGGGTTGATATAAGTAT

side2_core_49	CGATAGCAGCACTTTTTGTAAAACCGCCTCTTTTTCTCAGAAATC
side2_core_50	AATATAAAATTATTTGCACGTGCGATATTTTTCTTAGATTATACCGTCGCTA
side2_core_51	AGAAGGACTGAGACTATATCAAAGTACCGACAAAAGGTAACGCGCCT
side2_core_52	GTAAAACGACGGCCATTTTTTGCCAAGCTTTCAGGTTTTCCCAG
side2_core_53	TACTCAGCCCTCAGAACAGAGAGATAATTTTTCCACACGCCAGG
side3_core_1	TTGCGGGAAACGAGGGTAGCAACGAGTGAATAGATTTTAAGAACTGGC
side3_core_2	TCAAATATCCAGAACGAGTAGATTAATACCGATCGTCTGAAAT
side3_core_3	CAGGTCTTTCAAAAAGATTAAGAGCAGACCGGGAGATTTACCTTATGC
side3_core_4	ATCGTCATAAATATTCATTCAAAAATTACCAGACAGGAATTA
side3_core_5	TACAGGTAGGCTTTGACTTGCAGGGAGTTAAAGGAATTGCTATCATAA
side3_core_6	TTATTAATCAATAGGAGGTAAAGTTAACGAGAGGCTTTTG
side3_core_7	GCAACGACCAGTAATAACGCTCAAACGAACCAATTAGTCTTTAAT
side3_core_8	CATTTTGTATCATCATATGGGTCGAGGTTTTTCCGTTCATTTGC
side3_core_9	GGTCACGCTGCGGGCGCTATTTTTGGCGCTATAGATAA
side3_core_10	CCAACAGACGCCAGCCATTGCAATTTGAGTCCGAACG
side3_core_11	GTAAGAAAGCCGTCAGTTGAAAGCCCGGGTAGTTTCCTGAACATACG
side3_core_12	TTAATTCGATGATATCAAACCCTCAATCAATTGAGATGGAGCCTC
side3_core_13	TCTAAAATATCTGTTGCGTCCGTGTTTAATTGTAGTAAATTGGGCTGCTCACAA
side3_core_14	GAATCAGAGTTTTTGGGAGCTAAACAGGAGGGCAAGTGTAG
side3_core_15	CCCTCAAATAAAATTCAAATTGTAAACGTTAATTTAAAAG
side3_core_16	GTCAGTATTAACACCGCCTTTTTTCAACTTGTAGCAATACTTCT
side3_core_17	TTCCCAATTCTGCGCAGCCCTAAAACATCGCCATT
side3_core_18	ACATTTCCCATAAATGAATCCCAAAAGAGTTAAAT

side3_core_19	CGCCAAAGACGATAAAAACCAAAATAGACGCAGAAAAC
side3_core_20	CAGAACGATCAACTTTAATCATTGAGCTCAACAGCTTCAA
side3_core_21	GTCGGGGTCATTGCAGGCGTTTTTTTTCGCACTCTACGGTGGTG
side3_core_22	ACAGGCGGCGCGGTCCTACATTTGTAGATTAGTACGTGGC
side3_core_23	TCAACGTAACAAAGCTGCTCATTCGCTACAGA
side3_core_24	TGTACATCGACATAATTTTTAAAATCCCGTAAAACGCCAGCAGT
side3_core_25	TGCATCAGACGATCCATGTAAAGCGGTCCACGAATCATGG
side3_core_26	GCGGTTGCCTGGTTTGCCCCTTTTTGCAGGCGAAAATCGCCTGGCC
side3_core_27	TGTTCTGCAGATACATAAGAAAGACTGAGAATGA
side3_core_28	TGCCGTTCCGGCAAACCTTTAGTTCGACAACTCGTAGCACTAAATCGGA
side3_core_29	AACAACCCGTCGGATTCTCCGTGAGAATAGACAGAGGGGCCCTCGTT
side3_core_30	CAGCTTTCATCAACATTAAATGTGAGCGAGTCAGCTCAT
side3_core_31	CAGAAACGACTTGTAGAATTTTTGTCAGCGTGGTGCCATCCCA
side3_core_32	GAATAATACAGTTTCAGCGGAGTGGGAACAAACGGCGGATTGACCGT
side3_core_33	CCAGCGGTGCTTTTTGGTGCCCCCGGTATTTTTTGGGTAAAGGTTT
side3_core_34	CGCAACCATTTTTCTTACGGCTGGAGGTCTGTTGCCCTGCGGCT
side3_core_35	CGCTGAGGGGACTAAAGACTTTTTTACCCAAAGACGTTGGGA
side3_core_36	TGAGGATCGAATTGAGAGTTGGCAATGAAAAATCTAAAGCATCACCTAACAG AG
side3_core_37	CTCACAGTGTTTCTGCACAACTAAAGGATTTA
side3_core_38	AGTCACAATTTATTTACATTGGCAGACGCTCATGGAAA
side3_core_39	TATTTTTATTCTGGGAAGTATTAGACGTTATGCTGATCGTGCC
side3_core_40	TGCGGCGGCCGGGTCAGTCCAGCATCAGCTCGATAACGGAACGTGCCG
side3_core_41	TTTTTAACTATTTTGTTGCTTTAAATTCACC

side3_core_42	TGCAGCAAGCCTGGGGTGCCTAATGAGTGAGCTTTTTAACTCACATTAATTG
side3_core_43	TGTGAAATTGTTATCCATCTGGTCGAAGGTTAGTGAGGCGACAGACAA
side3_core_44	AGCCGGAAGCATAAAGGCGCAGTGGAATTCGTGGTATGAGGCCGTTTT
side3_core_45	CCTGTAGCCAAAAATAATTCGCGTAAATTAAATCCTTTGCAACATTAT
side3_core_46	GAAAGATTAGTAAGAGCAATGCTTTCGAGGTG
side3_core_47	CAGGAAAAACATTTACAAACAATGATGAAGACGCCAT
side3_core_48	CTGAGAAGATTAACCGAGTGCCACGTTTTTTGAGAGCC
side3_core_49	AAGGGATTTTAGTTTTTCAGGAACGGTACGCCTTCACC
side3_core_50	CATATCCAGAACAATTTTTTTTACGATAGAACCTTTTTTTCTGATCGG
side3_core_51	TACCTACATTTTGAAAGGGACGAATGGCTCGCTTAGGAGCACTACAGCACG
side3_core_52	AAATAGTTTGACCATCATCCAATAA
side3_core_53	TATGCAACTAACAGTTGAAGCGAACGAAGCCC
side3_core_54	AGCAGCAAAATCAACAGCCGATTATCATAGCTCCGAGCTCTCACTGCG
side3_core_55	GCGCGAACTGATAGAAGATAATGCTGAACCTCAATTTTAAA
side3_core_56	CCATATAAAGTACGGTTGAATATAATGCTGT
side1_hub_1	CGTTATACATTTCATCTTCTGACCTAATAGAAAAATCGCAAGACAATTACC
side1_hub_2	GCGAACACTCATCTTTAATAAACAAACATCAAGAAAACAAAATTA
side1_hub_3	AAGAATACACTAAAGAGTTTGAAATACCGACCGTGTGATAAATAA
side1_hub_4	AATCAATATGAGCAAAATGGAAACCAAAATCGTTGGGAAG
side1_hub_5	ATTATTTATTTTAGAATCCAAGCCTGTTTAGTATCATATG
side1_hub_6	GGCGTTAAATAAGGACCCCCAACAGCCCTGAGTTTCG
side1_hub_7	TTTGTTTAAGCGCATATGTGACCAAGTTAAGTACATA
side1_hub_8	ATTACATTTAACATTGTCGTCAGCGCCATCTTCTGGT

side2_hole_1	TGGGCGGTTGATCAAGTTTTTTGTCCGTGAACCAAGA
side2_hole_2	CCAGTAATAAGAGATCAAAATATAAAAACAGAAATAA
side2_hole_3	GTAACAGTAAGTTTATAAAATAATACAATAGAAGGCATTTTCGAG
side2_hole_4	ACCCTAAAGGGAGCCCCCGAAAGCGACCAACGTCGTTGTTCCGTG
side2_hole_5	TTCAGGTTCCGCCACGAACCTACCCCTCAAGATGAAAGTA
side2_hole_6	AGGTGGAGTAACGTCAAGAAATTGCGTAGATT
side2_hole_7	ATGCCAACGGCACACTGGTAGTTTGGACCGAAATCCGTGCTTT
side2_hole_8	TTCAGCAAATCAACCTGTCGGCAACAGCTGATTGCCCAGAATCCCTCGTTA
side3_hole_1	TGAATTAGGAATACAGCATCGGTCGTCACCCTCAGCAG
side3_hole_2	TGCCAGTTTGGTAATAGTAAAATTTTAGTTTTGCAAG
side3_hole_3	AAAGCCGCACATCCTCTCGCTGGCAGCCTCCGGGTGCTGCTACCGGGG
side3_hole_4	AGCGGATCAAACTTAAATTTCTGCCTGGCCTTGCCAGAGC
side3_hole_5	AGGCTTGCCCTGACGAGAAACACCGAAAGACCACATTCAACTAATTC
side3_hole_6	GAACAACTAAAGGCCGCTTCGAGGCATCATCAGTT
side3_hole_7	GTTTTTTCCACGGTCAGGCCAGAACGCCTGTGCACTCTGTTTCCACAC

Table S3 | Staple sequences triangular monomer 2.

Triangular monomer making up the protruding spikes of the DNA origami nanoshell.[3]

**Name**	**Sequence**
side1_core_1	GGAGGTGTGGTTGCGGACGCAGAAAACGGATAGTTGGGTA
side1_core_2	GTCAAAGGCAGTTTGGGTAGAACGGTAGGGGGTTTCTGCC
side1_core_3	CCACAAGATTAAGCAAATCAGATAGAGGCGTTTTTTTAGCGA

side1_core_4	CTCAGAGCATAAAGCTTAAGAAAAGTAAGCAGATAGCCG
side1_core_5	CTCCGGCTTAGGTTTTTTGGGTTATATAACGAATTATC
side1_core_6	ATCCCATCATCGGCTGACCGACAATTAGGCAGAGGCATT
side1_core_7	GAGCACATCCTCATAACGACCGCAAGAGCCGCACCAGTTGGG
side1_core_8	AGAGAGAAATTGAGTTAAGCCCAAGAGATAAC
side1_core_9	AAATCAAGATAATTATTCATTTTTTTCAATAACATAAGTCAGAGG
side1_core_10	TCATTGCAAGTCTCTGTGGTGCTGCGGCCAGAATGCCAT
side1_core_11	ATTAACACCGCTTCTGCTCATTTGCAGCGGGGGTCTGGTC
side1_core_12	CCAAAAGAACAACGCGGTCCGTTAAGGATTGCCGTGTACCA
side1_core_13	GGCATTCCAAGAACGGGTCAGTACCATCACCCAAATC
side1_core_14	ATGCGTTATACAAATAGAACGCTAGAAGGCTTATCCG
side1_core_15	AATCAATACTAATTTAAATGCAGAACGCGCCTGTTTATCATAAAGT
side1_core_16	AAAACGACGTTGGCAAATCAACAGAATCAATATCTGGTCA
side1_core_17	GTAATTGAAAGTTTTTCAAGCAAGACCAAGTA
side1_core_18	TTAACGTCTTTTTAAAATGAAGGGAGCCCCCTTTTTATTTAGAGCTTGGTTTTT AT
side1_core_19	CTCAACAATTTTCATCGTAGCGCTACGTGA
side1_core_20	TCACGGTCATACCTAAAGCCAACGTTCGAGCC
side1_core_21	GCCGTTTAGCAGCAGAACGTGCGATGCTAACGTGG
side1_core_22	AGCTATCTTACCGAAGCCCTTTTAAATCGGT
side1_core_23	GGGTTGAGTGTTGTTCGTATTCTATCTTACCATCAGGAATCATTACC
side1_core_24	TATATGTAGAATTTATCAAAATCATTTTTTGGTCTGAG
side1_core_25	GCGCTAATGCGCCCAATAGCACCTGAGCAAAAGA
side1_core_26	AGATGATGGCTCTTTAGGAGCACTAACAACTATTTTTTAGATTAGAGCCGTC

side1_core_27	ATCCTATCAGGGCGATGGCGGGTAAAGTTAAACCGGACTTAACAAGAG
side1_core_28	ACGCCAACCGCACTCTAGAAACCTGAAAAATAAACCCTC
side1_core_29	CATGTAATAAGGTAAAGTAATTCTGTCTTTTTAGACTTTCATCT
side1_core_30	CCTGAGAGATCAAAAATAATTCGCCGCCAGCTCGCCATGTTT
side1_core_31	TTCGCACTGTTTCCTGAACAAGAAAAATAATGGCCAGT
side1_core_32	TTTTGAATGGTTTTTATTAGTCTAGTATTTTTAAGAACTCAAACT
side1_core_33	TTCCGGCAGTAAAAAAAATGCCAATTACGGCTAGCTGTT
side1_core_34	GGGATAGCGGAACGCCTCTGGAGCAAACAAGAAAAGGCCG
side1_core_35	TCTCACGGTACATCGACATGGAGAGGGTAGCT
side1_core_36	GCGCGCCTGTGCAAATAAGAGAATAACAATAGATAAGACCTGCAGCCA
side1_core_37	AGACTACCGAGTGAATAACTTTTTTTGCTTCTGTAAAAATCAA
side1_core_38	TCTTTCCTTATCGCTCAACAGTGGCCAAGCTACGTTGT
side1_core_39	CCACGCTGAGAGCCAGGTGAGGCGGTATTAACCGTTTTTGTAGGGCT
side1_core_40	TCACCTTGCCGAACGAACCACCAGAGGACGCAAATTAACCGTTG
side1_core_41	AACAACATGTTCAGCTCGAGCATGTTTTTAACCAAATATCCTAAAGCA
side1_core_42	GCGGTGCTGTCACTCGGGCGCCCAGCATCCGCCAG
side1_core_43	CGGTGCCCTCGTTAACGGCATCACCACGGGACAGCGGTTTGTTA
side1_core_44	TTAATGCGCGAACTGACTAAAATAAATTCATCCTGAACCT
side1_core_45	AATATACACGGGAGAATTAACTGTTTTTACACCCTGAACAAAAAACAGGGAA
side1_core_46	AATAAATAAATATAATCCTGTTTTTTTGTTTGGATTATATCATATT
side1_core_47	AGCCTGTTTAGTTTTTTTCATTAATTGAGATTTTTTCGCCAAATA
side1_core_48	TATATGTATCGAGAATGGGGTCGCAGAAGATAAAACAGAGCAGCAAA
side1_core_49	AATCAGTGTTTTTGGCCACCGAGTAAAAAAACATCACTTGCCTG

side1_core_50	GGCGAAAGGGGGATGTGCTGCAAGAACCAATA
side1_core_51	ACGCCAGGGTTTTCCTGATAATCATCAAACTTAAATCTG
side2_core_1	TTTTTCATAGGTTTAGGCCCGTATAAACAGTTTTGACAGGTCTTTGAC
side2_core_2	TTTGAAAGAGTTAACCCTCGTTTACCAGACGACGAT
side2_core_3	ACCGTCGCCCTGAGATAGCATTACGGCGGATTGACCGTAAGTTTGAG
side2_core_4	CACTACGGGGTTGCCCTGATAGCTGCATTAATGAATCGGCCTAACCGA
side2_core_5	TTCCATTATAAATTGGGTCAGGACACAGGTAGAGGTCTTTA
side2_core_6	CCACGCAGTGCCGGAAACCAGGCTCCGGCACCGCTTCTG
side2_core_7	GATACCGATAGTTGCGCCGACAAAGGCTGAGTAATGC
side2_core_8	CTTGCTTTCGAGGTGAATTTCTTATACTCAGG
side2_core_9	GAACAAAACATCCAATAAATCTGATATTTTTATTAATGCCAAAGAGACAGT
side2_core_10	AGATAAGGCACCAACCTCTGCTCATGTGTACAGAGCAACACTATCATGAGG
side2_core_11	TACAAGCTGATGAACGTCAGTGAAACACAACTAATCGCCAAAAGGAA
side2_core_12	ATTCTACTAAAATACACGAAAATCCTGTTCAGCCTCCGGCCA
side2_core_13	CGGAACAAGATTTACACCAGAAACAAAGAAAACGAAAGCGCG
side2_core_14	ATCAGTTGACATTATTGTTGGGAAGAAAAATGGGAGTT
side2_core_15	GTCTGGCCTTTTTTTCTGTAGCCAAAGGTTTTTATCAGGTCATTG
side2_core_16	GCTTTCATCAACATTAGCGCAACTGAATTTGTGGAAGATC
side2_core_17	GGAATACCACATTTACGAGCCGGAACCTGTCGTGCCAAAACGAACTAA
side2_core_18	AAAATCCCTGGCATGATTAAGACTTTTTCCTTATTACGCAGTAACGGAATAC
side2_core_19	TACTCATTTGGGGCGCGACGGAGATTTGTATTGACCAACTTAGTTTG
side2_core_20	ATGTTAGCAAACGTAGAATAGTAGGAGTTGCAGCCCTTCA
side2_core_21	TTACGGGAATCAACGTACGAGTAGAACGGGTAAAAGGCCG

side2_core_22	TGAAATTGGCCTGGGGTGCCTAATGAGTTTTTGAGCAGACGATCCAGCGCAG
side2_core_23	GCAAAATCTTAGCTATATTTATAACCACGG
side2_core_24	TAAAGGTGAAAATCCGCGACCTGCATTGATAAATCCGCCTCC
side2_core_25	TCCTGTGCCGCCTGGGTATTGGGGGGACGACGCCAGCTT
side2_core_26	ATCCCCTCCACACATAAGAGACGGGCAACGGTCACGGGCATCA
side2_core_27	GGGTACCTCCACGCTGGTTACAGAAAAGGTTTGGTGT
side2_core_28	GTAACAGTTACCGCCACCCTCAGATTTATCAGGACAGCATCG
side2_core_29	AACCGTGCATCTGCCATGGGATAAGCTGATTGCAAGCGG
side2_core_30	CGGTTTGCAGGTTTCTGCACTCCAGACAGTATCGGCCTCATCTCCGTGG
side2_core_31	ATTTTGATGAGAGATAGACTTTTTTTCTCCGTGGTGAAACGTACAG
side2_core_32	GAGCTCGATTCGCGTCCGTGAGCATCAAAAGAAATCG
side2_core_33	TCAGCAAACCTGCATCTAACTCACATTTTTTAATTGCGTTGCGCTC
side2_core_34	CATCAATATTTTTGATATTCAACCGTTCTCGTGAGAGATCTACA
side2_core_35	AGCACGCGTGCCTTTTTGTTCATTCGTAATTTTTTATGGTGGCGG
side2_core_36	TCCCAATTCTGCGAACCCTTATAACTCCTCACAGGTGCCCCAGCAGG
side2_core_37	CGAGATATCCACTATTATTTTCGTCTCTTTTTTCGCGCAATAAT
side2_core_38	GAGGAAGTCACTAAAACACAAGCGTCATACAT
side2_core_39	TGGTGTGTGGGTCACTGTTGCCCTGTTTTTGGCTGGTA
side2_core_40	TTGCTCGTCATATTTTTACATCCCTTACACTCGGCGAA
side2_core_41	ACGGTCAATCATAAGGGAGCATAGGCATTATACCAAGAGGCA
side2_core_42	ACCGAACCATCGCCTTCGCAAATAGAAAATTCATATGGTTTACC
side2_core_43	AACGCGCGTGGTTTTTAGTGTAAATTATCCGCTCACAAT
side2_core_44	ACCAGTCCCGGTTGGGAAGGGCGATCGGTGCGTTTTTGCCTCTTCGCTATTA

side2_core_45	CTTTTCACCAGTGGCCGCATCGTTATATTCGACCATCGC
side2_core_46	GTCATAGCTGTGTCGACCCCAGCGTGGCTGACTTACCCAATAGCGTC
side2_core_47	TTCATCGGCATTTTCGAGCGCCAAAGACAAAAGGGCGAC
side2_core_48	ATGGGTAAGTGGTGCCATCTTTTTCACGCAACCAGCCGGCAGC
side2_core_49	CGCCAGGGGGGAGAGGACTGCCCGCTTTCCAGTCGGGAAAGCATAA
side2_core_50	AACAAAGATTAGCAAAATTTTTTAAGCAATAAAGCCAAATCAC
side2_core_51	GCAAAGACAAGGTGGCAACATATATGGTGATGGTGGTTCCGAATAGCC
side3_core_1	TAATTTGCCAGTTACAAAATAAAAAGGAGCGTTAGATTCGCCTGATT
side3_core_2	GGTAAATAGCGTCTTTCCGTAATCGCCAGCAAACCATCGATTTGCGGATGCTC CTT
side3_core_3	ATTCAACTTTAGCGTCAGTTTTTCTGTAGCGCGTTGCAGGTCA
side3_core_4	GAGAAAGGACAGGAACGGTACGCAACAATATTTTTCAGG
side3_core_5	ACAAATAACTCTGAATTTATTTTTCGTTCCAGTTCAAGGTTGAG
side3_core_6	GCTAACGACCGTTTTAAATATGCACATATAAC
side3_core_7	TTTGCACCCAGCTACAGAGGTTTTAATTACATAAAAATTACTAGAAAA
side3_core_8	GCGCATTAGATCGCGCAGAGGCGTAGATACCAAG
side3_core_9	GACGATTGTTTTTCCTTGATATTCACAACTGGTAATAAGTTTTA
side3_core_10	ATCGGCCTGTGGCACAGACAATATAATAGATACCTGATTATC
side3_core_11	TACCGCCAAGAACCCTTCTGACCTTAGACTTTACCACCAGAAGGAGCG
side3_core_12	AAAACGCTCTATAAAACAGAAATACCTTAGAATGAATTAC
side3_core_13	CAAATAAGAACGTGGCTTACAAAAGTAACAGTCAGTACATATCGTCGCTTTAA CA
side3_core_14	CACCCTCAGAGCCACCAAATCTCCAGCAACGGACAACTTT
side3_core_15	TTATCACCGTCACCGTTTTTCTTGAGCCATTGGAATTATTCAT

side3_core_16	GAATTAGAAGTAGCGATAAAGCCATAGCAGCAAGTTTCGTCACAGACAGTAAATGA
side3_core_17	AATCACCAGTAGCTCAACATGGAACGAGGCGCAG
side3_core_18	ATAGCAAGCCCAATAGAGGAATTGGTGAGAATAGAAAGGA
side3_core_19	ACCGCCACCCTTTTTCAGAACCGCACGGTTTTTGTCAGTGCCTTGA
side3_core_20	ATACATTTGAGGATTTAGAAGTATGAAAGCGT
side3_core_21	ACAAACAATTCGACAACTCGTATCTGGCCAAAATTATTTGCACGAAG
side3_core_22	AGCGCAGTATCCTCATCAGAATCAAGTTTGCCCGATTGAGGGAGGGAA
side3_core_23	ATAACGGCAATTTCATTTCCTTGAACCTCCGACCGTGTGAT
side3_core_24	ACGTTATACAACTAAGAACCCATAGACGTTAGCCCTCATCCTTTAAT
side3_core_25	AGCAATACATGTGAGAAGTACGGGGAAAGCCGGCGAAACGATTTTTTGT
side3_core_26	AATTTTTTCACGTTGAACCCTCATTTTGCTAAATGATACAAACGCCTG
side3_core_27	TATGGGATTTTCAGGGCAAACTACGGAGTGTA
side3_core_28	ATAATGGATTTAACGTCAGTTCTTTGATTAGT
side3_core_29	TTTTTCAAAATTTAAGACGCTAATTTTCAAGAAATCATCGGG
side3_core_30	CCTGACTATTATAGTCAGCTTCAATGAATTACCAACAGTT
side3_core_31	GGCTTTTGCTACAGAGGCTTTTTTTGAGGACTAAAGACCAGCGAAA
side3_core_32	GTAACGATCCAGTCACACGACCGTAACACTGAGCGGTCCCAATGAA
side3_core_33	TCTGACCTAATATATTTGCAAATCCAATCGC
side3_core_34	GAAATACAAATGCTTTAAAAGATTAAGAGGCGCGAGAAAAC
side3_core_35	AAATAAGGCGTTATATATTAATTGAGAAGAGAACCTACCACAAAGAA
side3_core_36	GCGGAATCGAGAATGACCATAAATCAATTTTTAATCAAAGATTC
side3_core_37	CAATACTTTGATAAGAGCGAACCATTTTCTGTCAGCGGACGAATAAT
side3_core_38	AGAATAACTTTTTTCAAGAAAGCTTTGATTGCTATTTATTTATCCCAATC

side3_core_39	ACACCGGAATCATAGAAGTTTTGCTGAATCCC
side3_core_40	CAGAGGGAGCTTAATTGCTACAAGGCCGGA
side3_core_41	GCTTTTGCTTAGAATATAATGCTGTAGCACCTGAATC
side3_core_42	ACAAAATTGAAGCCTTACCTCCCGACTTGCGGCTGGAAGT
side3_core_43	AAAATAAAATGTTTTTTAGACTAGGCATAGTAAGACCAGGC
side3_core_44	ATGCGCCGCTACAGGGAACGTGCTGGAGGCCG
side3_core_45	AGGGTTAGTCAATAGTAATGCTGATTAGTTAAGACGACAAT
side3_core_46	AATCATTGATCTTGACAAGTTTTACCGGATATTCACTTCATC
side3_core_47	ACTTCTGAAAGAATACTGCTGGTAATATCCAGCAGAATCC
side3_core_48	AGTTGATACCATTAGATACTCCATGTTATTTTTTAGTTGACGGA
side3_core_49	TACGGTGTATTTTATCCATTACCAGGCGCTAGGGCGCTGCGCGCTTA
side3_core_50	GAACGAGGGTAAAAAAAAGGCTCCAAAAGGAGTTTTTCTTTAATTGTATCGG
side3_core_51	GAGTAACATTATCATTAAGACAAATTAGATTAATGGTTT
side3_core_52	AGATGATGAAACAAACATAATGGAAAACCTTTTATGCGTAGA
side3_core_53	GCGATAGCGAAAAGCCCGAAAGACTTCAAATTAATTTT
side3_core_54	TGGCTTAGGGTAATAGCCAAAATAGCGAGAGTTCATTCACTAAAG
side3_core_55	AAGAGTAAGGTCATTGAATGGAATAGCATTCCACCAGTA
side3_core_56	TTATACCAGCTTGAGATGGTTTAATTTTTTTCAACTTT
side3_core_57	AACGATTAGAGAGTAAGTTAGCAACGTCAGAGCGGGAG
side3_core_58	CTTATGCGATTTTTTTTAAGAACTGGCTCAACCCTCAG
side3_core_59	CCAACAGGTCAGATATTTTGTCGAAAAGTTTTTGCCCGA
side3_core_60	ATCGCGTTAGCAAACTCAGAAAACGTCATAAATATTCAT
side3_core_61	AGACCGGATTAATTCGAGAAGCAAAGCGGATTGCATCAAAACAGTT

side1_hole_1	TTGAAAGGAATTGAGGAAGGTTATTAGCCCTAAAA
side1_hole_2	GCGAAAAACCGTAGATAATAAGAGCAAGAAACAATGAATATTTTAAATG
side1_hole_3	AAACAGGAAGATTAACTAGCATGTCAATCATA
side1_hole_4	CATCGCCATTAGAGTCTGTCCATCTGCCGTAA
side1_hole_5	TGTACCCCGGTCAGTCACGTTCAGAGGTGGAGCCGGATGCCGGCAATCCGC
side1_hole_6	AGCACTAAATCGGAACCCTAAAAATAGCAGCCTTTAC
side1_hole_7	CAATGCCTGAGTAATGTGTCTTTAGTGATGAACCATGGTGCTG
side1_hub_1	GATTCAAATACTTTTGAAATATTTAAATTGTAAACGT
side1_hub_2	TAATATTTTGTTACATTTTTTGCGATTAAACCTCACCGGAAACAA
side1_hub_3	TAAAGGGTGAGGAATCGATCGGTTGTGAAAAAGAGTATGAGCC
side1_hub_4	GAACGGTAAATCAGCTAAATTCGCATTAAATTAGAAAAGCCCCAA
side1_hub_5	ATCGTAAGTATAAGCCGGGAGAAGCCTTTATTTCAA
side1_hub_6	CGCAAGGATAAAACCCTCATAATAGCAATACTCCAAC
side1_hub_7	AGGCGGCAGGTAAAAAATAGAAATTTTACATTATGACCCTG
side2_hole_1	GCCACCACCCTCAGAGCCGCCTCTGAAACATG
side2_hole_2	CAAGGCAAAGATTACCAGAAGGAAACCGAGGAAACAAAGAAAC
side2_hole_3	AATAAGTTTATTTTGTCTCAGAACCGCCACCCTCAGA
side2_hole_4	GTAACAACCCGTAGCATACAGG
side2_hole_5	AAAGTATTAAGTGACAACAGTCGCTGAGGCTTGCACTACGTTAACGAGAAA
side2_hole_6	AAAGCGCCATTCGCCATTCAGGCTAATGTGAGCGA
side2_hub_1	AGGATTAGGATTACCTATTATACCAGAACAAACAAAG
side2_hub_2	AAATACGAGCCCGGTTCGGAAGCGGGGTTAAATCACCGGA
side2_hub_3	ACCCCGCCACCCACAATCAATGGTCAATAACCTGTGAGTAGAT

side2_hub_4	CTGCCTATAATAGGTGGGGTTGATATAAGTATACTCCTCAAGAGA
side2_hub_5	AGTGCCGTCGAGATATCACCGAACAGCTTCTTTTGCGGGATCGTC
side2_hub_6	AGAGCCGCCATAATCATTGCTCAGTACCAGGCGGATA
side2_hub_7	TTTGCCATCTTTTCGCCAGCAAATGCCCCAAAGAATA
side2_hub_8	CACCACCCTCAGAGAGAGCCACCACCGGAACCCCTTATTAGCG
side3_hub_1	GATTTTAGAAGGGAAATGGTTGC
side3_hub_2	CTAAACATTCCTCGTTAGAATCAACGCTGCGCGTAA
side3_hub_3	CAGATTCACATGGAAAGGATTATTGGGACATTTAAATCCT
side3_hub_4	CTCAATCGTCTGAAATTACCTACACAACAGGAATTAAAGGAGAAACA
side3_hub_5	TTTGACGAGCACGTATCGCGTACTGAAAGCGACAGCCATA
side3_hub_6	TTGCGGAATATCAACAGAGATGCCATTGTTTTGACG
side3_hub_7	CCACCACACCCGCGCAAGTGTCCAGAGCCTTACCAAC
side3_hub_8	TCTTTCCGTACCAGTAATAAAATACATTGG

Supplementary references

(1) Hermanson, G. T. Bioconjugate techniques. *Elsevier/AP* 2013, Third edition., xvii, 1146 pages.

(2) Kick, B.; Praetorius, F.; Dietz, H.; Weuster-Botz, D. Efficient Production of Single-Stranded Phage DNA as Scaffolds for DNA Origami. *Nano Lett* 2015, *15*, 4672-4676.

(3) Sigl, C.; Willner, E. M.; Engelen, W.; Kretzmann, J. A.; Sachenbacher, K.; Liedl, A.; Kolbe, F.; Wilsch, F.; Aghvami, S. A.; Protzer, U.; Hagan, M. F.; Fraden, S.; Dietz, H. Programmable icosahedral shell system for virus trapping. *Nat Mater* 2021, *20*, 1281-1289.
